# Comparative single-cell multiplex immunophenotyping of therapy-naive patients with rheumatoid arthritis, systemic sclerosis, and systemic lupus erythematosus shed light on disease-specific composition of the peripheral immune system

**DOI:** 10.3389/fimmu.2024.1376933

**Published:** 2024-04-25

**Authors:** József Á. Balog, Ágnes Zvara, Vivien Bukovinszki, László G. Puskás, Attila Balog, Gábor J. Szebeni

**Affiliations:** ^1^ Laboratory of Functional Genomics, Institute of Genetics, HUN-REN Biological Research Centre, Szeged, Hungary; ^2^ Core Facility, HUN-REN Biological Research Centre, Szeged, Hungary; ^3^ Department of Rheumatology and Immunology, Faculty of Medicine, Albert Szent-Gyorgyi Health Centre, University of Szeged, Szeged, Hungary; ^4^ Department of Internal Medicine, Hematology Centre, Faculty of Medicine University of Szeged, Szeged, Hungary; ^5^ Astridbio Technologies Ltd., Szeged, Hungary

**Keywords:** rheumatoid arthritis, progressive systemic sclerosis, systemic lupus erythematosus, mass cytometry, autoimmunity

## Abstract

**Introduction:**

Systemic autoimmune diseases (SADs) are a significant burden on the healthcare system. Understanding the complexity of the peripheral immunophenotype in SADs may facilitate the differential diagnosis and identification of potential therapeutic targets.

**Methods:**

Single-cell mass cytometric immunophenotyping was performed on peripheral blood mononuclear cells (PBMCs) from healthy controls (HCs) and therapy-naive patients with rheumatoid arthritis (RA), progressive systemic sclerosis (SSc), and systemic lupus erythematosus (SLE). Immunophenotyping was performed on 15,387,165 CD45^+^ live single cells from 52 participants (13 cases/group), using an antibody panel to detect 34 markers.

**Results:**

Using the t-SNE (t-distributed stochastic neighbor embedding) algorithm, the following 17 main immune cell types were determined: CD4^+^/CD57^–^ T cells, CD4^+^/CD57^+^ T cells, CD8^+^/CD161^–^ T cells, CD8^+^/CD161^+^/CD28^+^ T cells, CD8^dim^ T cells, CD3^+^/CD4^–^/CD8^–^ T cells, TCRγ/δ T cells, CD4^+^ NKT cells, CD8^+^ NKT cells, classic NK cells, CD56^dim^/CD98^dim^ cells, B cells, plasmablasts, monocytes, CD11cdim/CD172dim cells, myeloid dendritic cells (mDCs), and plasmacytoid dendritic cells (pDCs). Seven of the 17 main cell types exhibited statistically significant frequencies in the investigated groups. The expression levels of the 34 markers in the main populations were compared between HCs and SADs. In summary, 59 scatter plots showed significant differences in the expression intensities between at least two groups. Next, each immune cell population was divided into subpopulations (metaclusters) using the FlowSOM (self-organizing map) algorithm. Finally, 121 metaclusters (MCs) of the 10 main immune cell populations were found to have significant differences to classify diseases. The single-cell T-cell heterogeneity represented 64MCs based on the expression of 34 markers, and the frequency of 23 MCs differed significantly between at least twoconditions. The CD3^–^ non-T-cell compartment contained 57 MCs with 17 MCs differentiating at least two investigated groups. In summary, we are the first to demonstrate the complexity of the immunophenotype of 34 markers over 15 million single cells in HCs vs. therapy-naive patients with RA, SSc, and SLE. Disease specific population frequencies or expression patterns of peripheral immune cells provide a single-cell data resource to the scientific community.

## 1 Introduction

Inflammatory, rheumatic, and systemic autoimmune diseases collectively contribute to a significant burden on healthcare systems. Treatments are only partially effective, and disease severity and therapeutic responses in individual patients are unpredictable. The complexity of systemic autoimmune disease (SAD) etiology, an incomplete list of causative agents, environmental factors, and polygenetic predispositions make both the diagnosis and clinical management of these pathologies difficult (1). The known fundamentals of the pathological mechanisms of these SADs are beyond the scope of our study; however, the latest findings have been reviewed elsewhere for rheumatoid arthritis (RA) (2), systemic sclerosis (SSc) (3), and systemic lupus erythematosus (SLE) (4). The differential diagnosis of spectrum disorders, such as SADs with similar signs and symptoms, including RA, SSc, and SLE, remains a major challenge for clinicians. The progression of these diseases is unpredictable, and if any damage is fatal or chronic, it can lead to a substantial combined impact on premature mortality (5, 6). Therefore, early diagnosis of SADs is highly important before irreversible damage to several organs, such as the joints, kidneys, and lungs, which are frequently involved in autoimmune attacks, develops. The introduction of disease-modifying antirheumatic drugs (DMARDs) with the high importance of biological DMARDs (bDMARDs) and the advent of targeted inhibitors have reached a breakthrough, leading to disease stabilization and improved quality of life (7–9). However, a lack of treatment response occurs in severe cases or therapeutic resistance can develop (10–12). Therefore, stratifying patients with clinically heterogeneous diseases, such as SADs, has become a novel approach to understanding the complexity of imbalances in immune homeostasis, molecular profiling, and the integration of multi-omics data. Analyzing the immunophenotype in early, untreated SADs may provide information for precision medicine approaches and may suggest diverse underlying pathology leading to similar phenotype. Mass cytometry has been used earlier studying a wide list of human spectrum diseases with deep insight into the heterogeneity of the immunophenotype (13, 14). In line with this assumption, the complex immunophenotyping of SADs can facilitate the prediction of the severity of the disease and therapeutic response, in addition to suggestions for future precision medicine. Mulhearn et al. reviewed the potential of peripheral blood immunophenotyping to predict therapeutic outcomes in response to biologics in RA (15). Papadimitriou et al. summarized the link between the innate and adaptive arms of the immune system in the pathological mechanisms of SSc in the context of the currently available treatment regimen (16). Nagafuchi et al. and Nakayamada et al. recently reviewed the influence of immunophenotyping on therapeutic strategy planning for SLE (17, 18).

The multiparametric immunophenotyping of peripheral immunity may assist in better understanding the pathobiology of SADs because the heterogeneity of inherent and external factors can influence the pathological mechanism and therapeutic response (19). An earlier immunophenotyping study published by Nagafuchi et al. reported a link between the HLA-DRB1 genotype and a higher frequency of peripheral memory CXCR4^+^CD4^+^ T cells in patients with RA (20). Furthermore, a multidimensional analysis of the peripheral immunophenotype of 311 patients with RA revealed that the expansion of effector memory follicular helper T cells (Tfh) correlated with disease activity (21). Bader et al. analyzed the peripheral blood of 20 therapy-naive RA patients using a 23-marker mass cytometry (CyTOF) antibody panel and reported the following markers: p-p38, IkBa, p-cJun, p-NFkB, and CD86 in the cells of both the myeloid innate and adaptive branches (memory CD4^+^ T cells) of the immune system as potential markers for discriminating patients with RA from healthy donors (22). Koppejan et al. used a 36-marker CyTOF panel for the immunophenotyping of treatment-naive patients with early ACPA^+^ (anti-citrullinated protein antibodies) and ACPA-RA and found a reduced frequency of CD62L^+^ basophils in patients with ACPA-RA (23).

Immunophenotyping of 20 systemic sclerosis cases using a 36-marker CyTOF panel revealed 18 significant alterations in peripheral blood mononuclear cells (PBMCs), highlighting the involvement of CD4^+^, CD8^+^, mucosal-associated invariant T cells, and B-cell subsets in pathogenic chronic inflammation (24). In a mass cytometric study by Kroef et al., hierarchical clustering of PBMCs from 88 patients with SSc was performed using a 34-marker antibody panel. They found altered cell populations in four clusters: cluster 1 (*n* = 16) with high CD16^+^ monocytes and low memory B-cell subsets, cluster 2 (*n* = 25) with increased classical monocytes, cluster 3 (*n* = 8) with higher memory B-cell counts, and cluster 4 (*n* = 37) with lower circulating classical monocyte counts (25). Multiparametric flow cytometric investigation of 88 patients with early SSc showed a decrease in CD8^+^ T cells and an expansion of CD28^−^ and CD319^+^ within the CD4^+^ subset in the SSc group compared with HCs (26). Agarbati et al. analyzed 46 patients with SSc using an eight-color FACS panel and showed a higher ratio of CD38^+^ T cells and CD4^+^CD25^+^FOXP3^+^ regulatory T cells in patients with SSc (27). Agarbati et al. also investigated the humoral arm of the adaptive immune system in SSc and found a higher frequency of CD24^high^CD19^+^CD38^high^ regulatory B cells, more circulating CD38^high^CD27^+^ plasmablasts, and peripheral CD138^+^CD38^high^ plasma cells than in HCs (27).

An early immunophenotyping study revealed reduced expression of CD3^+^ and CD4^+^ T-cell markers and increased expression of CD8^+^ cytotoxic T-cell and CD20^+^ B-cell markers in SLE based on traditional flow cytometry of 21 SLE patients vs. HCs (28). Later, Perry et al. showed a higher ratio of CD38^+^HLA-DR^+^ T cells in SLE in a flow cytometric study analyzing samples from 35 patients with SLE compared with samples from HCs (29). Lee et al. also used traditional flow cytometry to compare the peripheral immune signatures of 13 patients with SLE and nine HCs. They found 29 immune subsets discriminating SLE from HCs, with the emphasis on lower DC and NK cell ratios in SLE, but elevated CD8^+^ NK Treg cells in lupus (30). Recently, Sasaki et al. published the most comprehensive immunophenotyping of lupus in nine early and 15 established SLE patients compared with controls using two CyTOF panels measuring 38–39 parameters. Their key findings were an increased frequency of ICOS^+^Ki-67^+^CD8^+^ T cells, Ki-67^+^ regulatory T cells, CD19^intermediate^Ki-67^high^ plasmablasts, and PU.1^high^Ki-67^high^ monocytes in patients with early SLE (31).

In this study, single-cell mass cytometric immunophenotyping of over 15 million single cells was performed on PBMC samples of healthy controls (HCs, *n* = 13) and therapy-naive patients with RA (*n* = 13), SSc (*n* = 13), and SLE (*n* = 13) using an antibody panel detecting 34 markers. DMARDs can influence the peripheral immunophenotype. Therefore, we enrolled therapy-naive patients, which makes this study unique in the field of clinical rheumatology. Our aim was to decipher the complex alterations in the peripheral immunity in SADs and to reveal disturbances in immune homeostasis that may contribute to our understanding of the specific pathobiology of RA, SSc, or SLE.

## 2 Materials and methods

### 2.1 Human participants

Patients were recruited during visits to the Department of Rheumatology and Immunology at the University of Szeged. Healthy controls were voluntary staff members of the BRC or the University of Szeged. The participants were informed of the research by a physician. Written informed consent was obtained from all the participants, and the study was reviewed and approved by the independent ethics committee of the university. Details regarding the study design and handling of biological materials were submitted to the Human Investigation Review Board of the University of Szeged under the 149/2019-SZTE Project Identification code. Laboratory studies and interpretations were performed on coded samples with personal and diagnostic identifiers removed. The study adhered to the principles of the most recent revision of the Declaration of Helsinki.

### 2.2 Study design

Multiplex protein analysis of 52 drug-naive patients with SADs [RA (*n* = 13; median: 57 years; range: 29–73 years; **Supplementary Table 1**), SSc (*n* = 13; median age: 63 years; range: 29–75 years; **Supplementary Table 2**), and SLE (*n* = 13; median: 50 years; range: 20–72 years; **Supplementary Table 3**) patients and age- and sex-matched healthy controls (*n* = 13; median: 54 years; range: 22–77 years) was performed. We enrolled newly diagnosed drug-naive patients with RA, SSc, and SLE who had not received antirheumatic treatment, including non-steroidal anti-inflammatory drugs (NSAIDs), DMARDs, or glucocorticoids, until the time of blood sampling. Patients with RA were diagnosed according to the latest American College of Rheumatology/European League Against Rheumatism criteria (32) (**Supplementary Table 1**). Thirteen newly diagnosed patients who fulfilled the criteria proposed by the 2013 American College of Rheumatology/European League Against Rheumatism classification criteria for SSc were enrolled (33). Four out of 13 patients were further classified as having limited cutaneous SSc, and nine out of 13 were classified as having diffuse cutaneous scleroderma according to LeRoy et al. (34) (**Supplementary Table 2**). Patients with SLE who met the 2012 Systemic Lupus Collaborating Clinics (SLICC) criteria and had active, newly diagnosed SLE were considered eligible (35). Several clinical and immunological parameters were assessed at the time of SLE diagnosis (**Supplementary Table 3**). Healthy controls were age- and sex-matched to patients and had a negative history of rheumatic symptoms and negative status upon detailed physical and laboratory examinations. No comorbidities were detected in the patients or controls that could have influenced our investigation, nor did they take any medication that could have interfered with the measurements.

### 2.3 PBMC isolation

PBMCs were isolated as previously described (36). Briefly, after the collection of 20 ml of blood in an EDTA vacutainer (Becton Dickinson, Franklin Lakes, New Jersey, USA), PBMCs were purified using Leucosep tubes (Greiner Bio-One, Austria) according to the manufacturer’s instructions. If the pellet was light red, 2 ml of ACK Lysing Buffer (ACK) was added at room temperature (RT, 20°C) for 2 min. Samples were washed twice with 10 ml of PBS, and cell count and viability were checked using Trypan Blue. PBMCs were cryopreserved in stocks of 4 × 10^6^ cells in 1 ml of FCS (Euroclone, Milano, Italy) supplemented with 1:10 DMSO (Merck, Darmstadt, Germany) [v/v] in liquid nitrogen.

### 2.4 Cell preparation

Cells were processed for CyTOF as described previously by our group with minor modifications (37). Briefly, cryotubes were thawed in a 37°C water bath for 2 min, and cells were transferred into 14 ml of cRPMI at 37°C and centrifuged at 350*g* for 6 min at room temperature (RT). PBMCs were washed once more with 10 ml of cRPMI, cells were counted, and viability was determined by Trypan Blue exclusion. PBMCs including up to 2–3 × 10^6^ cells/sample were plated onto a 96-well repellent plate separately in 200 µl of cRPMI and rested overnight in an incubator with 5% CO_2_ at 37°C. The rested cells were collected and washed twice with Maxpar Cell Staining Buffer (MCSB; Fluidigm, now Standard BioTools, South San Francisco, California, USA).

### 2.5 Barcoding and antibody staining

Mass cytometry was performed as previously described by our group with minor modifications (38, 39). Briefly, cells were resuspended in 50 µl of MCSB supplemented with 1:20 v/v Human TruStain FcX Fc Receptor Blocking Solution (BioLegend, San Diego, California, USA) and incubated at RT for 10 min. Anti-CD45 antibody-based live cell barcoding was performed as described previously by Fish et al. (40). Without the washing step, 50 µl of different metal-tagged (^89^Y, ^106^Cd, ^114^Cd, ^116^Cd) CD45 antibodies (clone: HI30; Fluidigm) at a final concentration of 1:100 [v/v] per antibody were added separately and incubated at 4°C for 30 min. PBMCs were washed twice with MCSB and 1 × 10^6^ cells from all four samples were pooled into 100 µl of MCSB. Cells were stained with 1:100 [v/v] of five markers, CD32, CD47, CD98, CD172a, and CD335 (Fluidigm), and incubated at RT for 20 min in MCSB. PBMCs were diluted by 200 µl of MCSB and transferred into a single tube of Maxpar Direct Immune Profiling Assay (Fluidigm) and incubated at RT for 30 min. The panel of antibodies used is listed in **Supplementary Table 4**. Cells were washed twice with MCSB, prefixed with 1 ml of Pierce™ 16% formaldehyde (w/v) (Thermo Fisher Scientific, Waltham, Massachusetts, USA) solution diluted in PBS to 1.6%, and incubated at RT for 10 min. Stained and prefixed cells were centrifuged at 800*g* at RT for 6 min and resuspended in 800 µl of Fix & Perm solution (Fluidigm) supplemented with 1:1,000 [v/v] ^191^Ir-^193^Ir DNA intercalator (Fluidigm) for overnight incubation.

### 2.6 CyTOF data acquisition

CyTOF samples were acquired as described previously by our group with minor modifications (36, 41). Samples were washed three times with MCSB and filtered through a 30-μm CellTrics gravity filter (Sysmex, Görlitz, Germany), and the cell concentration was adjusted to 7 × 10^5^/ml in CAS (cell acquisition solution) for the WB injector. Finally, EQ four-element calibration beads (Fluidigm) were added at a 1:10 ratio [v/v] and acquired using a properly tuned Helios mass cytometer (Fluidigm). From the pooled samples, 1.2 × 10^6^ events (3 × 10^5^/individual PBMC) were collected to identify rare cell subsets. The generated flow cytometry standard (FCS) files were randomized and normalized with the default settings of the internal FCS-processing unit of the CyTOF software (Fluidigm, version:7.0.8493).

### 2.7 Data processing

The randomized and normalized FCS files were uploaded to the Cytobank Premium analysis platform (Beckman Coulter). Exclusion of normalized beads, dead cells, debris, and doublets and manual debarcoding were performed as described in **Supplementary Figures 1**, **2**. No significant differences in the cell counts between the examined groups were observed. FCS files with CD45-positive living singlets were exported and further analyzed in R. Compensation methodology, FlowSOM clustering, and dimensionality reduction were adapted from Crowell et al. (42). FlowSOM was chosen following the publication of Weber et al. about the unsupervised analysis of CyTOF data (43). Data analysis was performed as described by Nowicka et al. (44). Using the BioConductor CATALYST and FlowCore R packages, the FCS files were integrated, compensated, and transformed. After signal spillover compensation, the CyTOF marker intensities were inverse-hyperbolic sine-transformed (arcsinh) with cofactor 5. For the main population definition, we performed self-organizing map-based method metaclustering on the compensated and transformed files. We identified 17 main different metaclusters as different cell types that were separately subclustered in another round of FlowSOM. High-dimensional reduction and visualization were performed using the (t-SNE) algorithm/method. In total, 300,000 cells and 34 markers were used to create a t-SNE map of the human peripheral immune system. The event numbers in the identified main immune cell populations and in the immune cell-related metaclusters are listed in **Supplementary Table 5** for each human subject. The minimum criteria for the cell number for the 17 main immune cell populations was at least 150 cells in each of the 10 subjects from the 13 participants meeting at least one of the conditions (HCs, RA, SSc, or SLE). The minimum criteria for the cell number for the metaclusters to move forward with the analysis was at least 50 cells in each of the 10 subjects from the 13 participants meeting at least one of the conditions (HCs, RA, SSc, or SLE).

### 2.8 Statistical analysis

Median signal intensities, cell frequencies, and subpopulation frequencies were analyzed using GraphPad Prism 8.0.1. The normality of distributions was tested using the D’Agostino and Pearson test and passed if all the groups’ alpha values were <0.05. Normally distributed datasets were compared using ordinary one-way ANOVA or Brown–Forsythe ANOVA when standard deviations were not equal. For non-parametric analysis, the Kruskal–Wallis test was used. All significance tests were corrected for multiple comparisons by controlling the false discovery rate (FDR) using the two-stage Benjamini, Krieger, and Yekutieli approach, with an FDR cutoff of 10%. Differences were considered significant at *p <*0.05.

## 3 Results

### 3.1 Enrollment of therapy-naive SAD patients and the workflow of single-cell immunophenotyping

**Figure 1:**
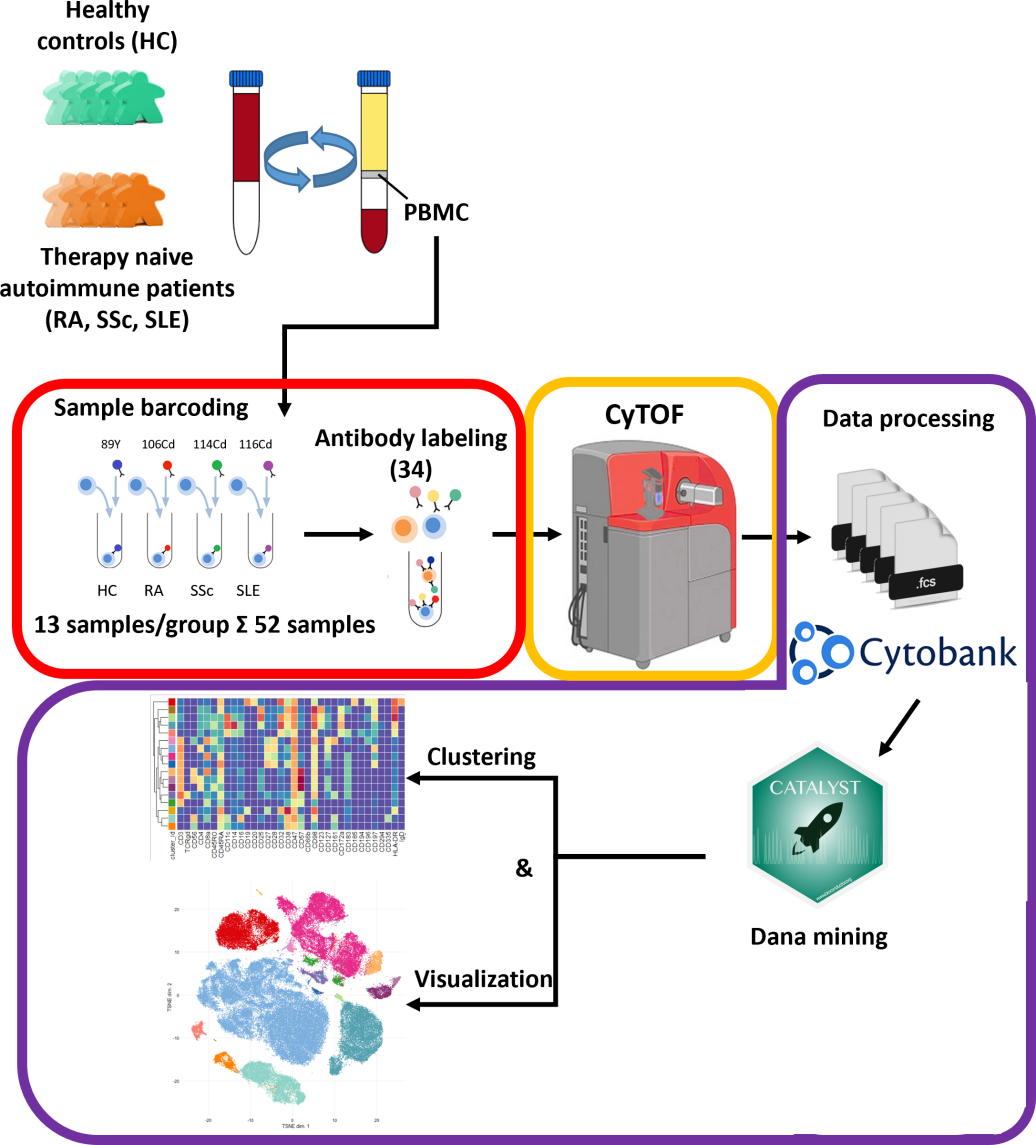
Schematic cartoon of the workflow of the study. Thirteen subjects were enrolled per group, namely, therapy-naive RA, SSc, and SLE patients and HCs. The PBMCs were purified from the peripheral blood by Ficoll-density gradient centrifugation. Immunophenotyping was performed using a 34-membered antibody panel optimized for single-cell mass cytometry. The PBMCs of four subjects were labeled separately with anti-CD45 antibodies conjugated with different metal tags. Subsequently, the cells of the four barcoded subjects were stained simultaneously in one tube. The CyTOF was performed by the Helios system. Data analysis was carried out using Cytobank Premium and Catalyst package in R software as described in the *Materials and methods* section.

Our aim was to perform single-cell immunophenotyping of SADs, namely, RA, SSc, SLE, and HCs. For better clarity, a schematic cartoon of the project workflow is summarized in **Figure 1**. The enrollment of therapy-naive SAD patients allowed unprecedented insight into the early stage of disease development without the masking effect of disease-modifying antirheumatic drugs following therapy.

### 3.2 Determination and characterization of the 17 main immune populations in HCs and therapy-naive patients with RA, SSC, and SLE

**Figure 2:**
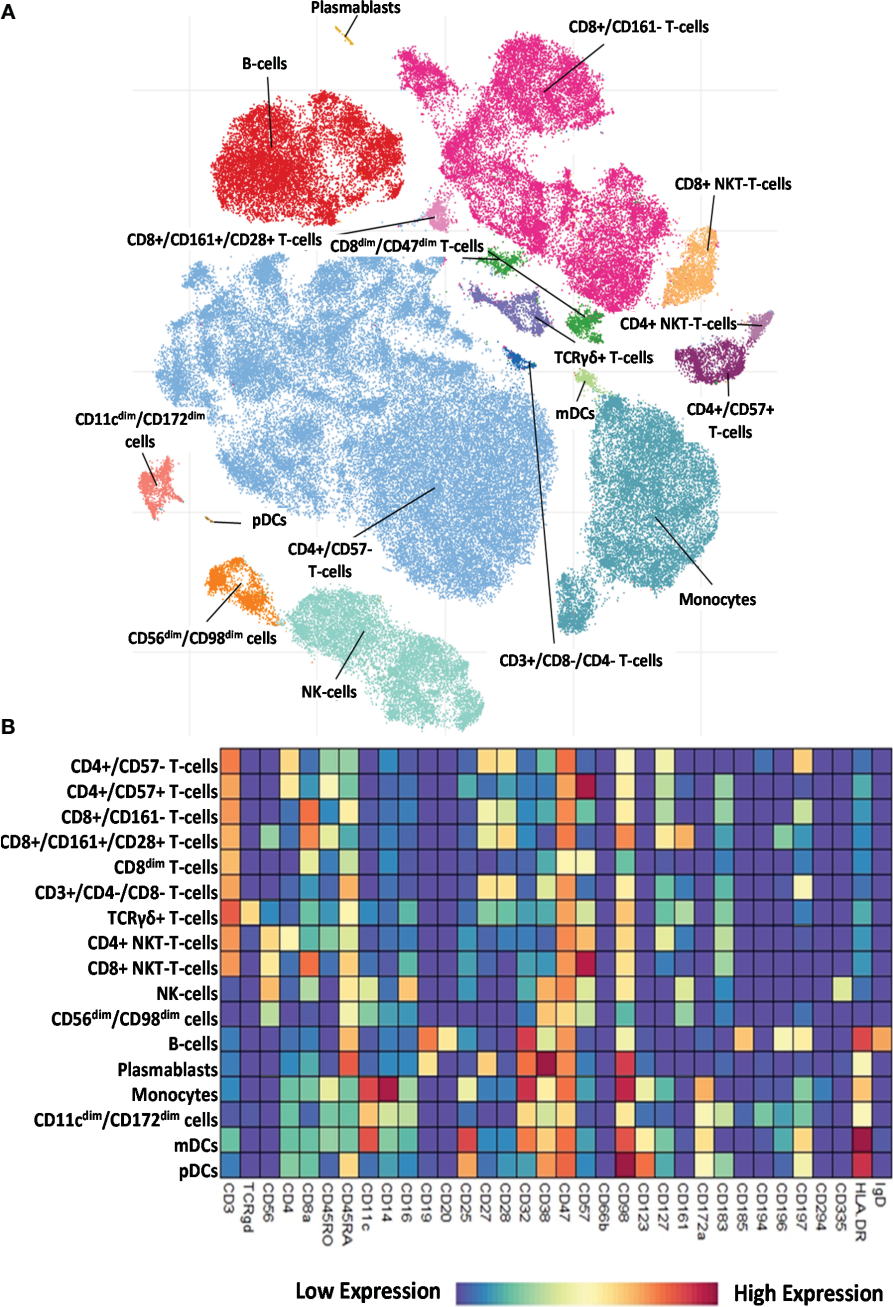
Single-cell immunophenotyping of leukocytes using 34 antibodies. **(A)** Representative viSNE diagram of the distribution of 17 main immune subsets with a single-cell resolution. Each dot represents one cell, and the size of one cloud is proportional to the size of that population. For visualization, the algorithm chose 3,000 cells randomly from each of the 52 samples. **(B)** The heatmap of the 17 main immune subsets showing their marker expression profile. Coloration indicates the intensity of the cell surface marker density. Dark red refers to high expression; dark blue refers to low expression.

The 34-marker antibody panel for the single-cell mass cytometric investigation and the subsequent FlowSOM analysis identified 17 immune cell types among the 15,387,165 cells from the 52 participants. Visualization of single-cell data delineated the 17 main immune cell populations in the viSNE plots (**Figure 2A**). The following seven T-cell types were identified: CD4^+^/CD57^−^ T cells, CD4^+^/CD57^+^ T cells, CD8^+^/CD161^−^ T cells, CD8^+^/CD161^+^/CD28^+^ T cells, CD8^dim^ T cells, CD3^+^/CD4^−^/CD8^−^ (DN = double negative) T cells, and TCRγ/δ T cells. The following four NK cell types were characterized: CD4^+^ NKT cells, CD8^+^ NKT cells, NK cells (classic NK), and CD56^dim^/CD98^dim^ cells. The following two B-cell types were analyzed: cells and plasmablasts. Three myeloid cell types were studied: monocytes, CD11c^dim^/CD172^dim^ cells, and myeloid dendritic cells (mDCs). Finally, innate lymphoid plasmacytoid dendritic cells (pDCs) were also involved in patient immunophenotyping. The expression profiles of the 17 immune cell populations for the 34 investigated markers are shown on a heatmap (**Figure 2B**), where data were aggregated from 52 FCS files (13 participants/group). This expression analysis supplemented the viSNE plot for the discrimination of the 17 immune cell populations, highlighting both common and cell-type-specific marker expression.

**Figure 3:**
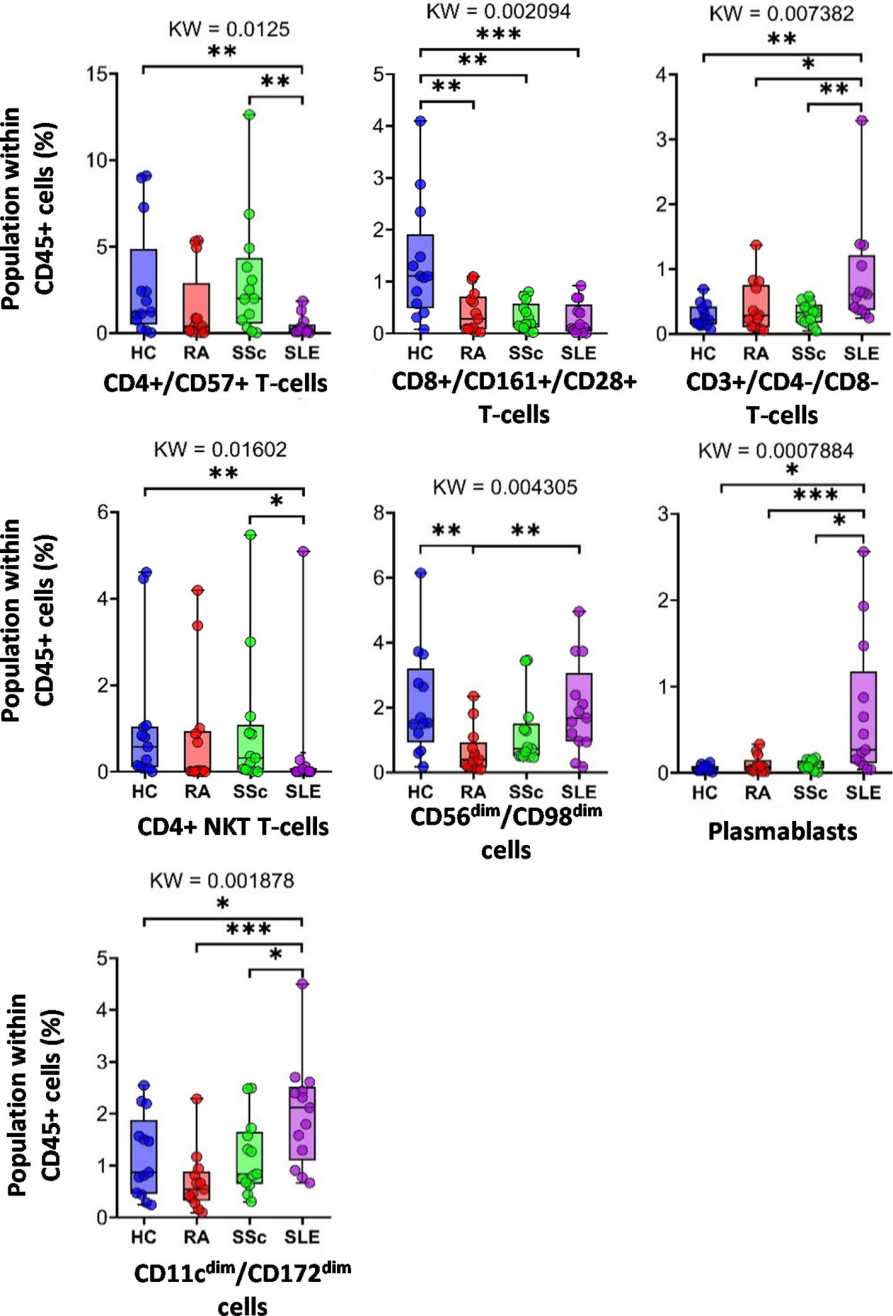
The percentage of the main immune subsets within the matured living peripheral CD45^+^ leukocytes. Only significant changes are shown here, and non-significant differences are illustrated in **Supplementary Figure 3**. The groups were compared using the Kruskal–Wallis (KW) test, and the results are shown on the top of each column bar. Significance was determined when the *q*-value of the false discovery rate (FDR) was below 0.1 and *p* < 0.05; *p < 0.05, **p < 0.01, ***p < 0.001. The values shown on the column bar from the bottom to the top: lower bar = minimum value, bottom line of the chart = lower quartile (Q1), middle line = median, top line of the chart = upper quartile (Q3), upper bar = maximum value.

Next, we examined the distribution of the identified peripheral immune cell types among the HC, RA, SSc, and SLE groups. Significant differences in the population percentages are shown in **Figure 3**. Seven populations showed significantly different frequencies: CD4^+^/CD57^+^ T cells, CD8^+^/CD161^+^/CD28^+^ T cells, DN T cells, CD4^+^ NKT cells, CD56^dim^/CD98^dim^ cells, plasmablasts, and CD11c^dim^/CD172a^dim^ cells. CD4^+^/CD57^+^ aging T cells showed the lowest frequency in the SLE group (0.402% in SLE vs. 3.089% in SSc or 2.819% in HCs). CD8^+^/CD161^+^/CD28^+^ mucosal-associated invariant T cells (MAIT) were at the highest frequency in healthy controls (1.351% in HCs vs. 0.405% in RA, 0.323% in SSc, and 0.286% in SLE). DN T cells showed the highest incidence in SLE (0.867% in SLE vs. 0.279% in HCs, 0.421% in RA, or 0.307% in SSc). CD4^+^ NKT cells were significantly decreased in SLE (0.432% in SLE vs. 1.083% in HCs or 0.968% in SSc). CD56^dim^/CD98^dim^ NK cells were reduced in RA (0.657% in RA vs. 2.133% in HCs or 1.967% in SLE). The percentage of plasmablasts was significantly higher in SLE (0.686% in SLE vs. 0.053% in HCs, 0.101% in RA, or 0.097% in SSc). CD11c^dim^/CD172^dim^ monocytes (with low expression of CD32, CD47, CD98, and HLA-DR) were also more prevalent in SLE (2.008% in SLE vs. 1.187% in HCs, 0.682% in RA, and 1.178% in SSc). The remaining 10 of the 17 main populations did not show differential distributions among the investigational groups. The distribution of these 10 populations is shown in **Supplementary Figure 3**.

### 3.3 Disease-specific expression intensities of single-cell mass cytometry data comparing peripheral immune cells in HCs, RA, SSc, and SLE

Characterization of the 34-marker expression of the 17 main populations is shown in the viSNE plots in **Supplementary Figure 4**. The areas of the t-SNE plots correspond to the main 17 immune subsets, as shown in **Figure 2A**. Next, the individual expression data (only significant changes among the four groups) were plotted on the scatter plots as follows: CD4^+^/CD57^−^ T cells, CD4^+^/CD57^+^ T cells, CD8^+^/CD161^−^ T cells (**Supplementary Figure 5**); CD8^+^/CD161^+^/CD28^+^ T cells, CD8^dim^ T cells (**Supplementary Figure 6**); DN T cells (**Supplementary Figure 7**); TCR γ/δ T cells, CD4^+^ NKT cells, CD8^+^ NKT cells (**Supplementary Figure 8**); NK cells, CD56^dim^/CD98^dim^ cells (**Supplementary Figure 9**); B cells and plasmablasts (**Supplementary Figure 10**); monocytes, CD11c^dim^/CD172a^dim^ cells (**Supplementary Figure 11**); and mDCs and pDCs (**Supplementary Figure 12**).

Here, we highlight the primary differences. Except for helper T cells, CD38 expression in all cell types was higher in at least one autoimmune disease than in HCs. In the case of DN T cells (**Supplementary Figure 7**), TCRγ/δ^+^ T cells, CD8a^+^ NKT cells (**Supplementary Figure 8**), NK cells (**Supplementary Figure 9**), and monocytes (**Supplementary Figure 11**), all three patient groups had significantly higher CD38 expression compared with HCs (in the case of NK cells, there was no significant difference between RA vs. HCs, *p* = 0.0681). The results were similar for the CD8a^dim^/CD47^dim^ population, with the addition of the SLE group showing significantly higher CD38 expression than the other two patient groups (**Supplementary Figure 6**). In the case of CD8a^+^/CD161^−^ cytotoxic T cells, the SLE group expressed significantly higher levels of CD38 compared with all the three other groups (**Supplementary Figure 5**), whereas in the case of mDCs, the difference between HCs and SLE was significant (**Supplementary Figure 12**). In patients with SLE, in contrast to CD38, CD45RA had the lowest expression in immune cells. We observed a significantly lower expression of CD45RA compared with the HC, RA, and SSc groups in the following cell types: CD4^+^/CD57^+^ T cells (**Supplementary Figure 5**), CD8a^dim^/CD47^dim^ T cells (**Supplementary Figure 6**), CD56^dim^/CD98^dim^ cells (**Supplementary Figure 9**), and B cells (**Supplementary Figure 10**). Comparing HCs vs. SLE, we detected significantly lower expression of CD45RA in CD8a^+^ NKT cells (**Supplementary Figure 10**), NK cells (**Supplementary Figure 9**), and pDCs (**Supplementary Figure 12**) in the SLE group. There was only one exception: CD45RA expression was higher in SLE and the other two autoimmune patient groups than in HCs in DN T cells (**Supplementary Figure 7**). In patients with SSc, the expression of the two markers was significantly higher than that in the other three groups: CD57 expression in CD4^+^/CD57^+^ T cells (**Supplementary Figure 5**) and CD16 expression in NK cells (**Supplementary Figure 9**). Patients with RA were also differentiated from the other conditions by significantly different expressions as follows: in CD11c^dim^/CD172a^dim^ cells, the expression of CD32 and CD98 was significantly higher than in the other three groups (**Supplementary Figure 11**). CD98 expression in CD56^dim^/CD98^dim^ cells was higher in the RA group than in the other three groups (**Supplementary Figure 9**). CD47 expression in CD11c^dim^/CD172a^dim^ cells was significantly higher (**Supplementary Figure 11**), whereas in CD8a^+^/CD161^+^/CD28^+^ T cells, it was significantly lower in patients with RA than in the HC, SSc, and SLE groups (**Supplementary Figure 6**). The expression of HLA-DR in TCRγ/δ^+^ T cells (**Supplementary Figure 8**), B cells (**Supplementary Figure 10**), and mDCs (**Supplementary Figure 12**) was also significantly lower in the RA group compared with the other three groups.

Taken together, 59 scatter plots demonstrated significant marker expression differences in the 17 main immune populations differentiating therapy-naive patients with RA, SLE, and SSc from HCs and between the SADs (**Supplementary Figures 5**–**12**). However, a detailed explanation of these data is beyond the scope of our research paper; rather, these **Supplementary Data** provide a resource and repository for the scientific community. Next, the authors preferred to thoroughly analyze and explain the unsupervised FlowSOM data of the subsequent analysis of the cell-type heterogeneity of the 17 main populations, the distribution of metaclusters (subpopulations), and significant differences in their marker expressions.

### 3.4 Characterization of the specific RA, SSc, and SLE differences in the single-cell immunophenotype of the subpopulations of the 17 main immune cell types of peripheral blood

**Figure 4:**
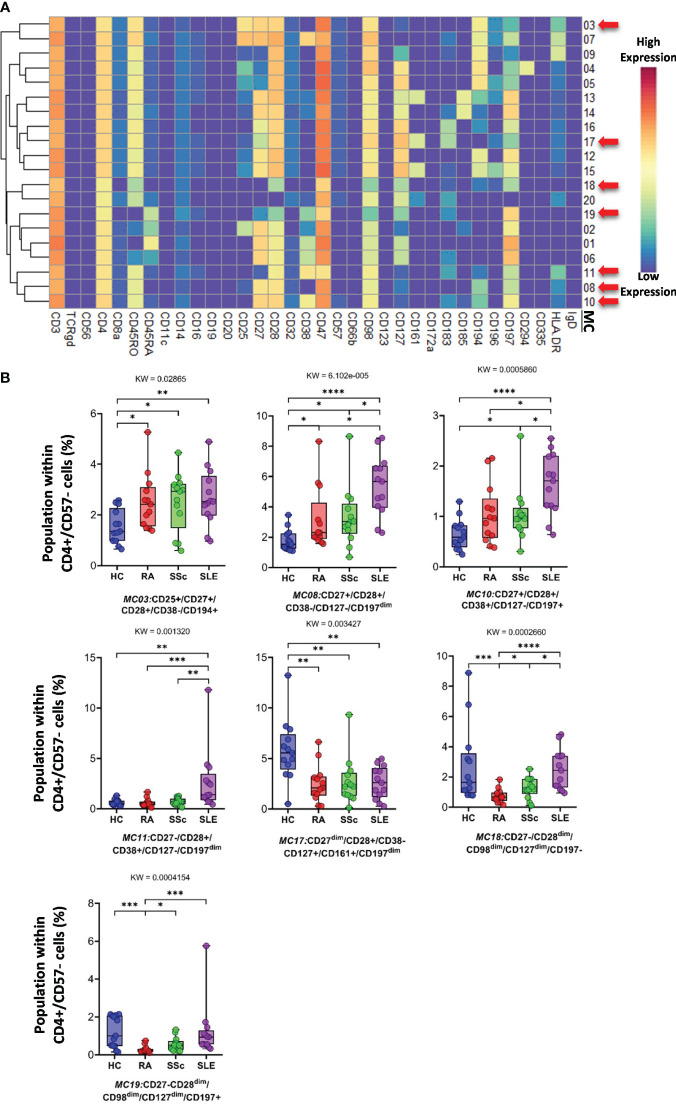
The subpopulations of CD4^+^/CD57^−^ helper T cells. **(A)** Marker expression heatmap of the CD4^+^/CD57^−^ helper T cells divided into 20 MCs by the FlowSOM algorithm. Coloration indicates the intensity of the cell surface marker density. Dark red refers to the highest expression; dark blue refers to the lowest expression. Red arrows highlight the MCs with significant differences among the studied groups. **(B)** Chart diagrams of the MCs with significantly different frequencies among the studied groups. The differences between groups were evaluated using the Kruskal–Wallis (KW) test, and the results are shown on the top of each column bar. Significance was determined when the *q*-value of the false discovery rate (FDR) was below 0.1 and *p* < 0.05; *p < 0.05, **p < 0.01, ***p < 0.001, ****p < 0.0001. The values shown on the column bar from the bottom to the top: lower bar = minimum value, bottom line of the chart = lower quartile (Q1), middle line = median, top line of the chart = upper quartile (Q3), upper bar = maximum value.

Analysis of FlowSOM metaclusters of mass cytometry data revealed intracell-type heterogeneity of each main immune cell type in therapy-naive cases of RA, SSc, and SLE vs. HCs. First, the *CD4^+^/CD57^−^ T cells* were divided into 20 subpopulations (MCs = metaclusters), and the heatmap of the marker expression profile of the MCs is shown in **Figure 4A**. Visualization and a viSNE map of the MCs of CD4^+^/CD57^−^ T cells are shown in **Supplementary Figure 13A**. The size of the MCs in the viSNE plot is proportional to the number of cells within an MC, and the proximity of the MCs is proportional to the common marker expression profile (**Supplementary Figure 13A**). The cell density plots highlighted HC and disease-specific MC distribution (**Supplementary Figure 13A**). Seven MCs (red arrows) showed significant differences within CD4^+^/CD57^−^ T cells (**Figures 4A, B**, **Supplementary Figure 13A**). One Treg subpopulation (CD4^+^/CD25^+^/CD45RA^−^/CD127^−^) and MC03 (CD25^+^CD38^−^CD127^−^CD194^+^) were the lowest in HCs (HCs: 1.554%; RA: 2.520%; SSc: 2.520%; SLE: 2.675%). One effector memory (T_EM_) T-cell (CD45RA^−^/CD197^−^) subpopulation, MC08 (CD4^+^/CD27^+^/CD28^+^/CD38^−^/CD127^−^/CD197^−^), was the lowest in HCs and the highest in SLE (HCs: 1.797%; RA: 3.156%; SSc: 3.320%; SLE: 5.396%). The MC10 (CD27^+^CD28^+^CD38^+^CD127^−^CD197^+^) and CD4^+^ central memory (T_CM_) T-cell (CD45RA^−^CD197^−^) subpopulations were the highest in SLE (HCs: 0.648%; RA: 1.040%; SSc: 1.049%; SLE: 1.634%). The other T_EM_ subpopulation, MC11 (CD27^−^CD28^+^CD38^+^CD127^−^CD197^−^), was also the highest in SLE, highlighting the discrimination from RA and SSc, not only from HCs (HCs: 0.624%; RA: 0.593%; SSc: 0.716%; SLE: 2.675%). MC17 (CD27^−^CD28^+^CD38^−^CD127^+^CD161^+^CD183^+^CD197^−^) was elevated in HCs (HCs: 5.793%; RA: 2.523%; SSc: 2.747%; SLE: 2.605%). Two populations (MC18 and MC19) were significantly lower in RA patients with a common lack of CD98, CD28, and CD27, and MC18 (CD27^−^CD28^dim^CD98^dim^CD127^−^CD197^−^) was the lowest in RA patients (HCs: 2.718%; RA: 0.749%; SSc: 1.278%; SLE: 2.514%). MC19 differed from MC18 in the expression of the CCR7 receptor (CD27^−^CD28^dim^CD98^dim^CD127^−^CD197^+^), which was significantly decreased in RA (HCs: 1.223%; RA: 0.277%; SSc: 0.570%; SLE: 1.227%).

**Figure 5:**
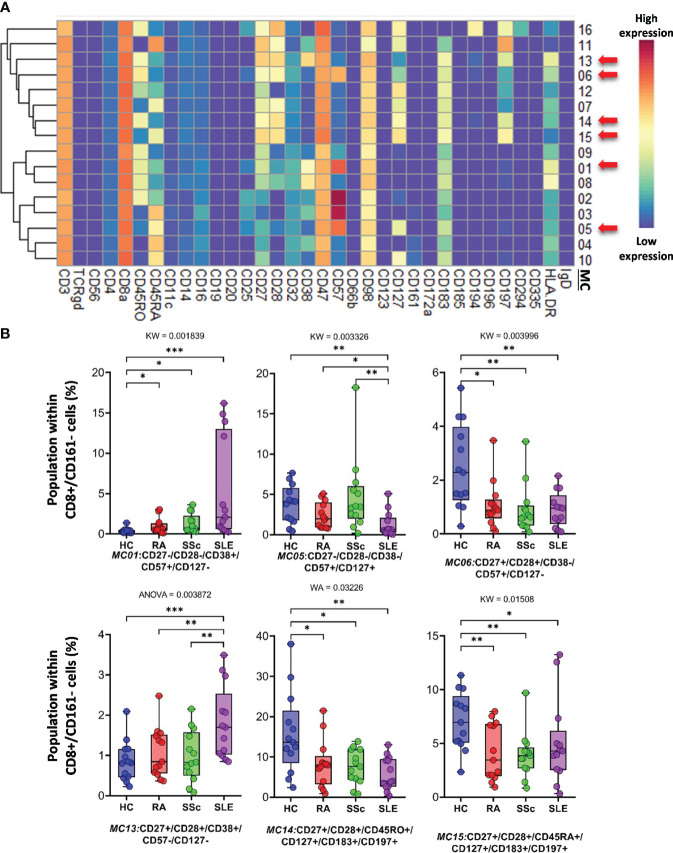
The subpopulations of CD8^+^/CD161^−^ cytotoxic T cells. **(A)** Marker expression heatmap of the CD8^+^/CD161^−^ helper T cells divided into 16 MCs by the FlowSOM algorithm. Coloration indicates the intensity of the cell surface marker density. Dark red refers to high expression; dark blue refers to low expression. Red arrows highlight the MCs with significant differences among the studied groups. **(B)** Chart diagrams of the MCs with significantly different frequencies among the studied groups. The differences between groups were evaluated using the Kruskal–Wallis (KW) test, Welch-ANOVA (WA), or one-way ANOVA (ANOVA), and the results are shown on the top of each column bar. Significance was determined when the *q*-value of the false discovery rate (FDR) was below 0.1 and *p* < 0.05; *p < 0.05, **p < 0.01, ***p < 0.001. The values shown on the column bar from the bottom to the top: lower bar = minimum value, bottom line of the chart = lower quartile (Q1), middle line = median, top line of the chart = upper quartile (Q3), upper bar = maximum value.

*CD8^+^/CD161^−^ cells* were divided into 16 MCs, and six MCs differed significantly from the other three groups (**Figure 5A** and **Supplementary Figure 13B**). The expression pattern of CD8^+^/CD161^−^ MCs is summarized on the heatmap (**Figure 5A**). The MC01 (CD27^−^CD28^−^CD38^+^CD57^+^CD127^−^HLA-DR^+^) was the highest in SLE (HCs: 0.408%; RA: 1.004%; SSc: 1.300%; SLE: 5.383%). MC05 (CD27^−^CD28^−^CD38^−^CD57^+^CD127^+^HLA-DR^−^), which differed in CD38^−^ and CD127^+^ from MC01, was the lowest in SLE (HCs: 3.803%; RA: 2.407%; SSc: 4.731%; SLE: 1.289%) (**Figure 5B**). The MC05 is a subpopulation within CD8^+^_TEMRA_: CD45RA^+^CD197^−^ (terminally differentiated effector memory cells re-expressing CD45RA). MC06 (CD27^+^CD28^+^CD38^−^CD57^+^CD127^−^HLA-DR^−^) differed from MC01 and MC05 in terms of CD27^+^CD28^+^ and CD38^−^CD127^−^ and showed the highest prevalence in HCs (HCs: 2.492%; RA: 1.083%; SSc: 0.921%; SLE: 0.910%) (**Figure 5B**; **Supplementary Figure 13B**). Similar to MC01, MC13 (CD27^+^CD28^+^CD38^+^CD57^−^CD127^−^HLA-DR^+^) was the highest in SLE (HCs: 0.866%; RA: 1.075%; SSc: 0.991%; SLE: 1.809%): CD27^+^CD28^+^ and CD57^−^. MC14 (CD27^+^CD28^+^CD45RO^+^CD127^+^CD183^+^CD197^+^) (HCs: 15.719%; RA: 8.301%; SSc: 7.556%; SLE: 5.911%) and MC15 (CD27^+^CD28^+^CD45RA^+^CD127^+^CD183^+^CD197^+^) were the highest in HCs (HCs: 7.087%; RA: 4.167%; SSc: 3.941%; SLE: 5.065%).

**Figure 6:**
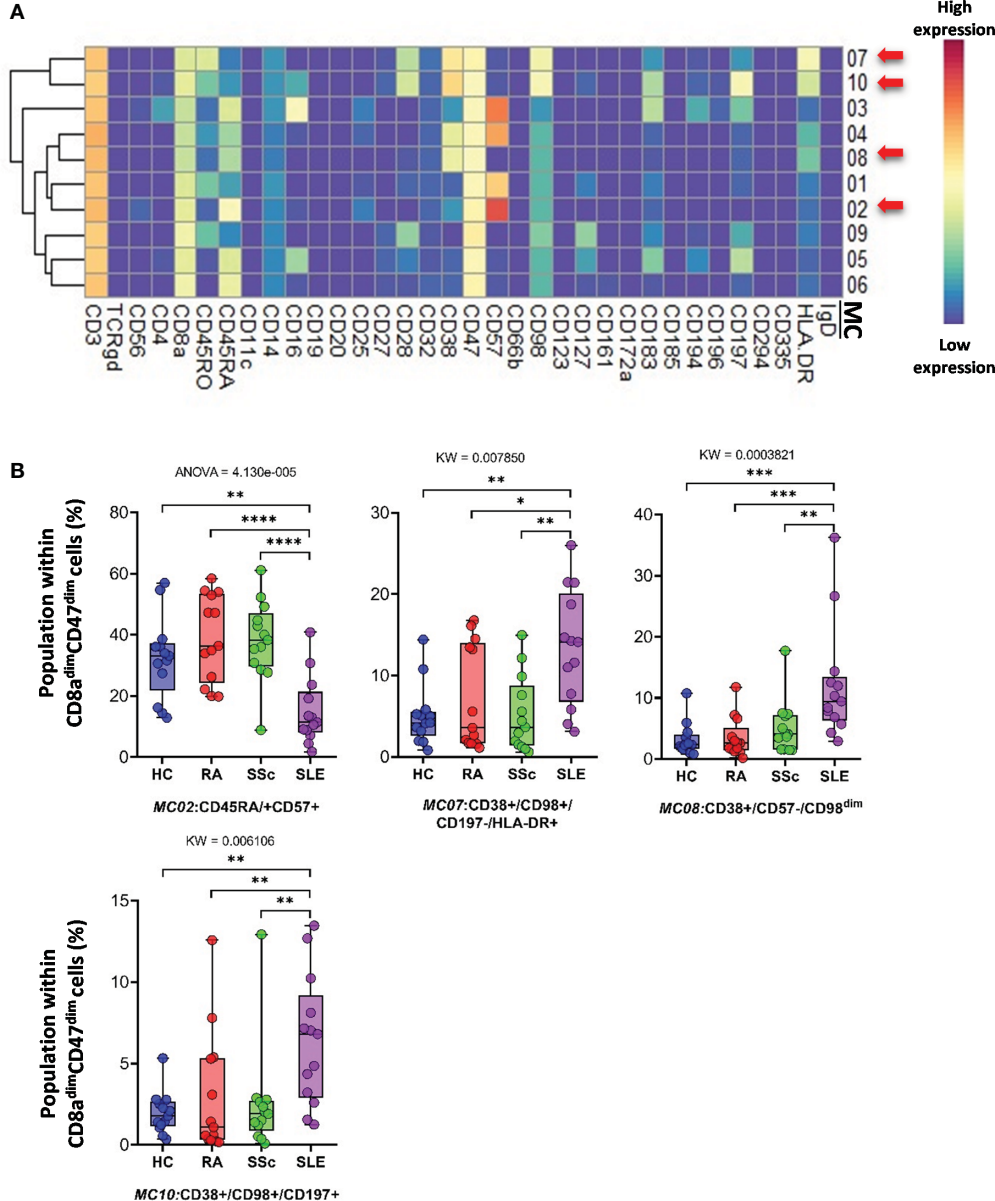
The subpopulations of CD8a^dim^/CD47^dim^ cytotoxic T cells. **(A)** Marker expression heatmap of the CD8a^dim^/CD47^dim^ helper T cells divided into 10 MCs by the FlowSOM algorithm. Coloration indicates the intensity of the cell surface marker density. Dark red refers to high expression; dark blue refers to low expression. Red arrows highlight the MCs with significant differences among the studied groups. **(B)** Chart diagrams of the MCs with significantly different frequencies among the studied groups. The differences between the groups were evaluated using the Kruskal–Wallis test (KW) or one-way ANOVA (ANOVA), and the results are shown on the top of each column bar. Significance was determined when the *q*-value of the false discovery rate (FDR) was below 0.1 and *p* < 0.05; *p < 0.05, **p < 0.01, ***p < 0.001, ****p < 0.0001. The values shown on the column bar from the bottom to the top: lower bar = minimum value, bottom line of the chart = lower quartile (Q1), middle line = median, top line of the chart = upper quartile (Q3), upper bar = maximum value.

*CD8a^dim^/CD47^dim^ T cells* represented 10 MCs, in which four MCs differentiated into HCs and therapy-naive RA, SSC, and SLE. A heatmap of the expression intensities of the 34 markers in CD8a^dim^/CD47^dim^ T cells is shown in **Figure 6A**. The viSNE diagram and cell density plots of CD8a^dim^/CD47^dim^ T cells are shown in **Supplementary Figure 13C**. MC02 (CD45RA^+^CD57^+^) cells in patients with SLE were min. half (or less) than those in the other three groups (HCs: 32.482%; RA: 39.018%; SSc: 38.138%; SLE: 14.912%) (**Figure 6B**). The other three MCs that dominated in SLE were MC07 (CD38^+^CD197^−^HLA-DR^+^) (HCs: 5.081%; RA: 7.228%; SSc: 5.179%; SLE: 13.378%), MC08 (CD38^+^CD57^−^CD197^−^HLA-DR^−^) (HCs: 3.209%; RA: 3.461%; SSc: 5.072%; SLE: 12.104%), and MC10 (CD38^+^CD197^+^) (HCs: 1.963%; RA: 2.981%; SSc: 2.501%; SLE: 6.404%) (**Figure 6B**).

**Figure 7:**
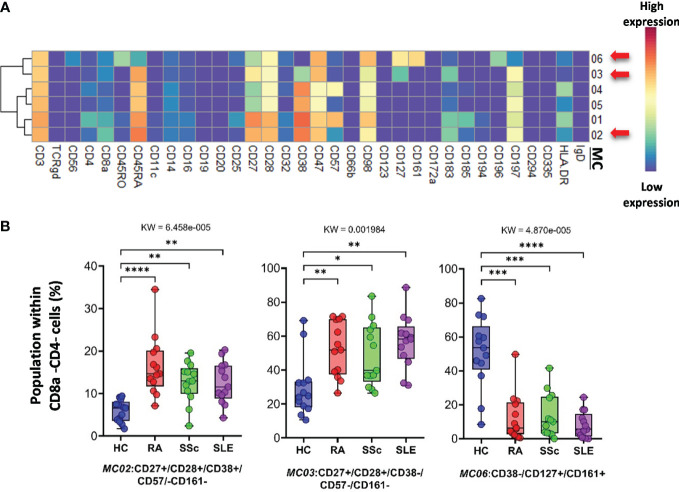
The subpopulations of CD8a^−^/CD4^−^ cytotoxic T cells. **(A)** Marker expression heatmap of the CD8a^−^/CD4^−^ helper T cells divided into six MCs by the FlowSOM algorithm. Coloration is proportional to the intensity of the cell surface marker density. Dark red refers to the highest expression; dark blue refers to no expression. Red arrows highlight the MCs with significant differences among the studied groups. **(B)** Chart diagrams of the MCs with significantly different frequencies among the studied groups. The differences between groups were evaluated using the Kruskal–Wallis test (KW), and the results are shown on the top of each column bar. Significance was determined when the *q*-value of the false discovery rate (FDR) was below 0.1 and *p* < 0.05; *p < 0.05, **p < 0.01, ***p < 0.001, ****p < 0.0001. The values shown on the column bar from the bottom to the top: lower bar = minimum value, bottom line of the chart = lower quartile (Q1), middle line = median, top line of the chart = upper quartile (Q3), upper bar = maximum value.

*CD3^+^/CD4^−^/CD8^−^ (DN) T cells* were divided into six subpopulations (**Figure 7**; **Supplementary Figure 14A**). Red arrows on the expression heatmap indicate MCs that differentiated SADs from each other (**Figure 7A**). MCs were also observed in the viSNE and cell density plots (**Supplementary Figure 14A**). MC02 (CD27^+^CD28^+^CD38^+^CD57^−^CD161^−^), similar to CD38^+^CD8 T cells, was the lowest in HCs (HCs: 5.868%; RA: 16.456%; SSc: 12.705%; SLE: 12.568%) (**Figure 7B**). The CD38^−^ MC03 (CD27^+^CD28^+^CD38^−^CD57^−^CD161^−^), similar to MC02, was also the lowest in HCs (HCs: 29.748%; RA: 52.147%; SSc: 48.745%; SLE: 54.712%). In contrast to MC02 and MC03, MC06 (CD38^−^CD127^+^CD161^+^) was the highest in HCs (HCs: 50.881%; RA: 12.540%; SSc: 13.452%; SLE: 8.154%) (**Figure 7B**).

**Figure 8:**
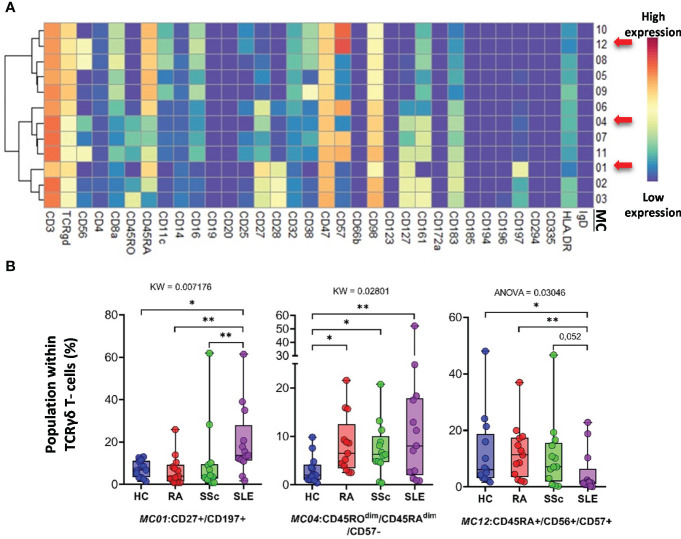
The subpopulations of TCRγδ T cells. **(A)** Marker expression heatmap of the TCRγδ T cells divided into 12 MCs by the FlowSOM algorithm. Coloration indicates the intensity of the cell surface marker density. Dark red refers to high expression; dark blue refers to low expression. Red arrows highlight the MCs with significant differences among the studied groups. **(B)** Chart diagrams of the MCs with significantly different frequencies among the studied groups. The differences were evaluated using the Kruskal–Wallis (KW) test or one-way ANOVA (ANOVA), and the results are shown on the top of each column bar. Significance was determined when the q-value of the false discovery rate (FDR) was below 0.1 and *p* < 0.05; *p < 0.05, **p < 0.01. The values shown on the column bar from the bottom to the top: lower bar = minimum value, bottom line of the chart = lower quartile (Q1), middle line = median, top line of the chart = upper quartile (Q3), upper bar = maximum value.

The FlowSOM algorithm revealed 12 MCs in the *TCRγ/δ T-cell* compartment. The heatmap of the expression of 34 markers in the TCR γ/δ T-cell population is shown in **Figure 8A**. The viSNE and cell density plots of the frequency of MCs are shown in **Supplementary Figure 14B**. One population of naive (CD27^+^CD45RA^+^) TCRγ/δ T cells, the MC01 (CD27^+^CD197^+^), was the highest in SLE (HCs: 7.254%; RA: 6.665%; SSc: 10.563%; SLE:19.745%) (**Figure 8B**). One effector memory TCRγ/δ T-cell population was the lowest in HCs (HCs: 3.024%; RA: 8.301%; SSc: 7.405%; SLE: 12.351%). In contrast to MC01, MC12 (CD45RA^+^CD56^+^CD57^+^) was significantly lower in SLE patients (HCs: 11.691%; RA: 12.172%; SSc: 10.995%; SLE: 4.929%).

**Figure 9:**
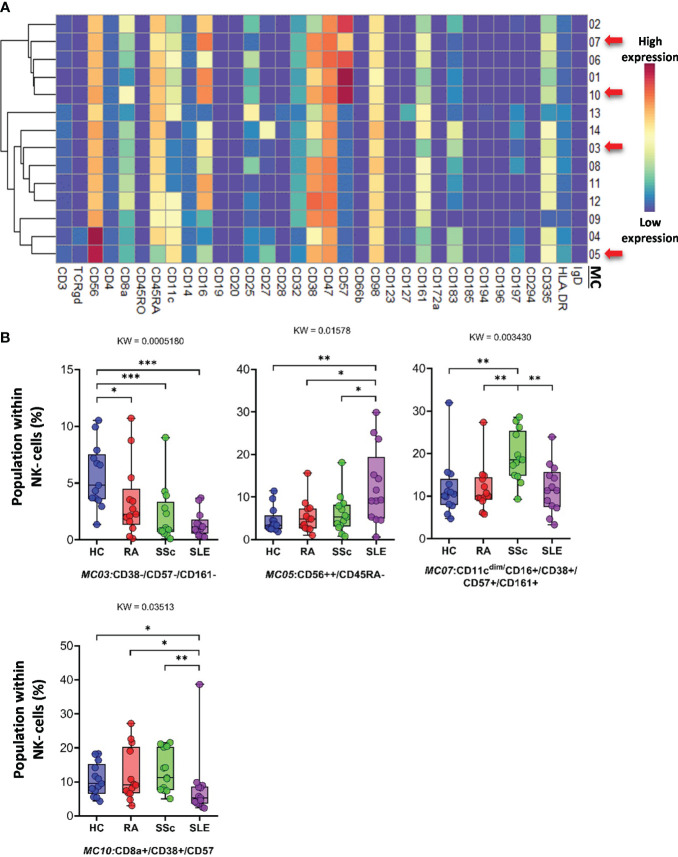
The subpopulations of NK cells. **(A)** Marker expression heatmap of the NK cells divided into 14 MCs by the FlowSOM algorithm. Coloration indicates the intensity of the cell surface marker density. Dark red refers to high expression; dark blue refers to low expression. Red arrows highlight the MCs with significant differences among the studied groups. **(B)** Chart diagrams of the MCs with significantly different frequencies among the studied groups. The differences between groups were evaluated using the Kruskal–Wallis (KW) test, and the results are shown on the top of each column bar. Significance was determined when the *q*-value of the false discovery rate (FDR) was below 0.1 and *p* < 0.05; *p < 0.05, **p < 0.01, ***p < 0.001. The values shown on the column bar from the bottom to the top: lower bar = minimum value, bottom line of the chart = lower quartile (Q1), middle line = median, top line of the chart = upper quartile (Q3), upper bar = maximum value.

Classical *CD3^−^/CD56^+^ NK cells* represented 14 MCs. Clustering of MCs based on the expression patterns of 34 markers is shown in **Figure 9A**. The viSNE and cell density plots of the MCs are shown in **Supplementary Figure 14C**. MC03 (CD38^−^CD57^−^CD161^−^) was almost two times higher in HCs than in RA and SSc and three times higher in HCs than in SLE (HCs: 5.624%; RA: 3.384%; SSc: 2.131%; SLE: 1.379%). MC05 (CD56^bright^CD45RA^−^) cells were more than double in the PBMCs of SLE patients compared to those in the other three groups (HCs: 4.559%; RA: 5.354%; SSc: 6.066%; SLE: 12.501%) (**Figure 9B**). MC07 (CD16^+^CD38^+^CD57^+^CD161^+^) showed double the frequency in SSc compared with HCs, RA, or SLE (HCs: 11.864%; RA: 11.810%; SSc: 19.294%; SLE: 11.811%). In contrast to MC05, MC10 (CD8a^+^CD38^+^CD57^+^) was the lowest in patients with SLE (HCs: 10.642%; RA: 12.004%; SSc: 13.058%; SLE: 8.152%).

**Figure 10:**
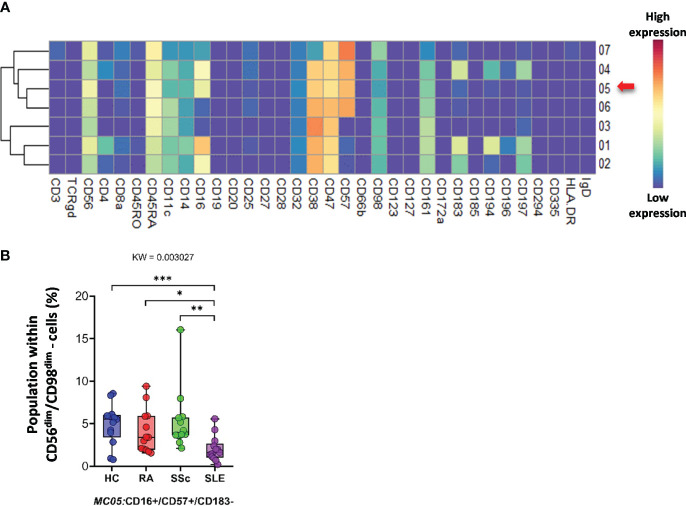
The subpopulations of CD56^dim^/CD98^dim^ cells. **(A)** Marker expression heatmap of the CD56^dim^/CD98^dim^ cells divided into seven MCs by the FlowSOM algorithm. Coloration indicates the intensity of the cell surface marker density. Dark red refers to high expression; dark blue refers to low expression. Red arrows highlight the MCs with significant differences among the studied groups. **(B)** Chart diagrams of the MCs with significantly different frequencies among the studied groups. The differences between the groups were evaluated using the Kruskal–Wallis (KW) test, and the results are shown on the top of each column bar. Significance was determined when the *q*-value of the false discovery rate (FDR) was below 0.1 and *p* < 0.05; *p < 0.05, **p < 0.01, ***p < 0.001. The values shown on the column bar from the bottom to the top: lower bar = minimum value, bottom line of the chart = lower quartile (Q1), middle line = median, top line of the chart = upper quartile (Q3), upper bar = maximum value.

*CD56^dim^/CD98^dim^ NK cells* were divided into seven MCs. The expression profiles of the 34 markers are shown in **Figure 10A**. The viSNE plots of the seven MCs and cell density plots are shown in **Supplementary Figure 15A**. Only one MC, MC05 (CD16^+^/CD57^+^/CD183^−^), showed a significant difference, and the percentage of cells in MC05 was almost half of that in the HC, RA, and SSc groups (HCs: 4.921%; RA: 4.077%; SSc: 5.249%; SLE: 2.049%).

**Figure 11:**
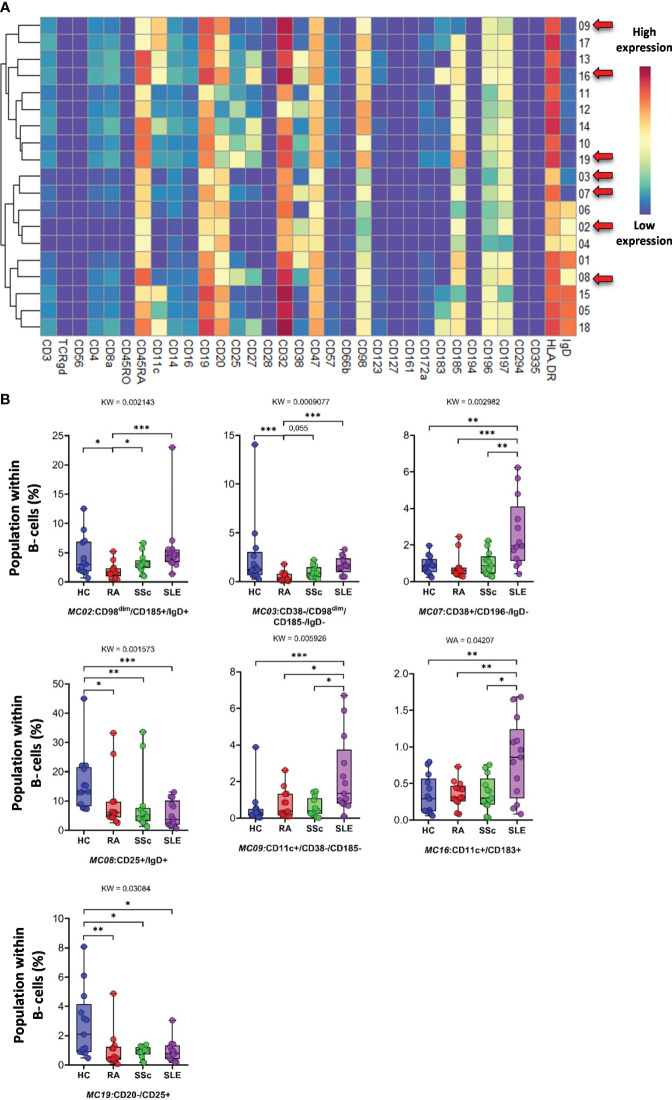
The subpopulations of B cells. **(A)** Marker expression heatmap of the B cells divided into 19 MCs by the FlowSOM algorithm. Coloration indicates the intensity of the cell surface marker density. Dark red refers to high expression; dark blue refers to low expression. Red arrows highlight the MCs with significant differences among the studied groups. **(B)** Chart diagrams of the MCs with significantly different frequencies among the studied groups. The differences among the groups were evaluated using the Kruskal–Wallis (KW) test, and the results are shown on the top of each column bar. Significance was determined when the *q*-value of the false discovery rate (FDR) was below 0.1 and *p* < 0.05; *p < 0.05, **p < 0.01, ***p < 0.001. The values shown on the column bar from the bottom to the top: lower bar = minimum value, bottom line of the chart = lower quartile (Q1), middle line = median, top line of the chart = upper quartile (Q3), upper bar = maximum value.

*CD19^+^ B cells* showed high heterogeneity, with 19 identified MCs. Seven MCs differentiated patients with SADs from each other or from HCs. The expression of 34 markers among the 19 MCs is shown in **Figure 11A**. The viSNE and cell density plots of 19 MCs of conventional peripheral B cells are shown in **Supplementary Figure 15B**. Two MCs were the lowest in RA, MC02 (CD98^dim^/CD185^+^/IgD^+^) (HCs: 4.320%; RA: 1.871%; SSc: 3.223%; SLE: 5.761%) and MC03 (CD38^−^/CD98^dim^/CD185^−^/IgD^−^) (HCs: 2.606%; RA: 0.490%; SSc: 0.975%; SLE: 1.662%). The number of MC07 (CD38^+^/CD196^+^/IgD^−^) B cells was significantly higher in the SLE group (HCs: 0.914%; RA: 0.802%; SSc: 0.988%; SLE: 2.638%) (**Figure 11B**). The number of MC08 (CD25^+^/IgD^+^) B cells was double in HCs than in RA or SSC and three times higher in HCs than in SLE (HCs: 16.239%; RA: 9.337%; SSc: 8.581%; SLE: 5.493%). The two CD11c^+^ B-cell populations were significantly higher in the SLE group than in the other three groups: MC09 (CD11c^+^/CD38^−^/CD185^−^) (HCs: 0.562%; RA: 0.785%; SSc: 0.571%; SLE: 2.312%) and MC16 (CD11c^+^/CD183^+^) (HCs: 0.337%; RA: 0.356%; SSc: 0.364%; SLE: 0.838%). The CD20^−^/CD25^+^ MC19 B cells were the highest in HCs (HCs: 2.772%; RA: 0.998%; SSc: 0.897%; SLE: 0.949%).

**Figure 12:**
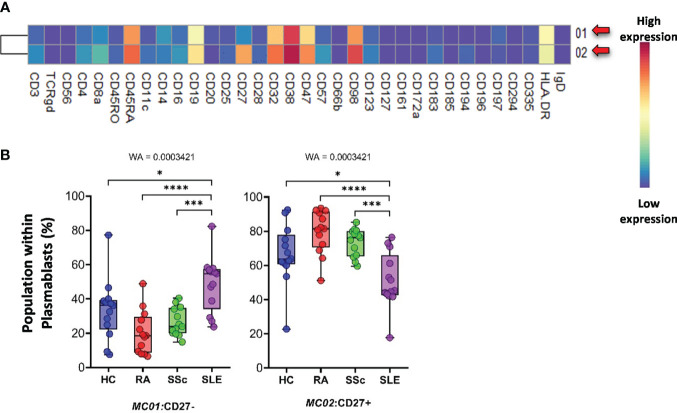
The subpopulations of plasmablasts. **(A)** Marker expression heatmap of the plasmablasts divided into two MCs by the FlowSOM algorithm. Coloration indicates the intensity of the cell surface marker density. Dark red refers to high expression; dark blue refers to low expression. Red arrows highlight the MCs with significant differences among the studied groups. **(B)** Chart diagrams of the MCs with significantly different frequencies among the studied groups. The differences between the groups were evaluated using the Welch-ANOVA test (WA), and the results are shown on the top of each column bar. Significance was determined when the *q*-value of the false discovery rate (FDR) was below 0.1 and *p* < 0.05; *p < 0.05, ***p < 0.001, ****p < 0.0001. The values shown on the column bar from the bottom to the top: lower bar = minimum value, bottom line of the chart = lower quartile (Q1), middle line = median, top line of the chart = upper quartile (Q3), upper bar = maximum value.

The *plasmablasts* in the peripheral blood represented two MCs depending on their expression (MC02) or lack of CD27 (MC01) (**Figure 12A**). The viSNE and cell density plots of plasmablasts are shown in **Supplementary Figure 15C**. The MC01 (CD27^−^) was the highest in SLE (HCs: 33.527%; RA: 20.424%; SSc: 26.482%; SLE: 48.871%). In contrast, MC02 (CD27^+^) was significantly lower in patients with SLE (HCs: 66.472%; RA: 79.575%; SSc: 73.517%; SLE: 51.128%) (**Figure 12B**).

**Figure 13:**
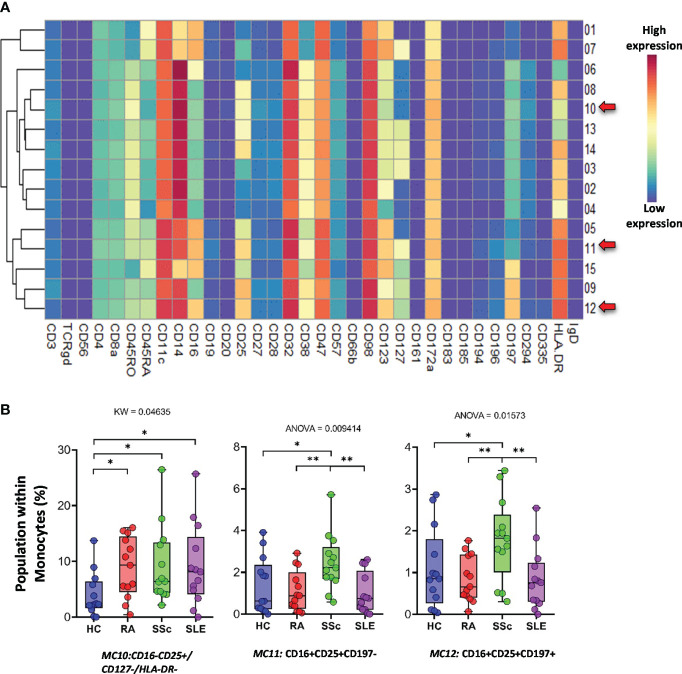
The subpopulations of monocytes. **(A)** Marker expression heatmap of the monocytes divided into 15 MCs by the FlowSOM algorithm. Coloration indicates the intensity of the cell surface marker density. Dark red refers to high expression; dark blue refers to low expression. Red arrows highlight the MCs with significant differences among the studied groups. **(B)** Chart diagrams of the MCs with significantly different frequencies among the studied groups. The differences between the groups were evaluated using the Kruskal–Wallis (KW) test or one-way ANOVA (ANOVA), and the results are shown on the top of each column bar. Significance was determined when the *q*-value of the false discovery rate (FDR) was below 0.1 and *p* < 0.05; *p < 0.05, **p < 0.01. The values shown on the column bar from the bottom to the top: lower bar = minimum value, bottom line of the chart = lower quartile (Q1), middle line = median, top line of the chart = upper quartile (Q3), upper bar = maximum value.

The *monocytes* were classified into 15 types of MCs using the FlowSOM algorithm (**Figure 13A**). A heatmap of the expression profiles is shown in **Figure 13A**. The distribution of MCs in the viSNE and cell density plots is shown in **Supplementary Figure 16**. One group of classical monocytes (CD14^++^/CD16^−^) and MC10 (CD16^−^CD25^+^CD127^−^HLA-DR^−^) were higher in RA, SSc, and SLE than in HCs (HCs: 3.977%; RA: 8.902%; SSc: 9.207%; SLE: 9.176%) (**Figure 13B**). Two transitional monocyte populations (CD14^++^/CD16^+^) showed a higher percentage of SSc with double frequency than the other three groups: MC11 (CD16^+^CD25^+^CD197^−^) (HCs: 1.369%; RA: 1.101%; SSc: 2.457%; SLE: 0.994%) and MC12 (CD16^+^CD25^+^CD197^+^) (HCs: 1.081%; RA: 0.844%; SSc: 1.786%; SLE: 0.845%).

## 4 Discussion

To the best of our knowledge, this is the first study to characterize the detailed immunophenotypes of patients with three different newly diagnosed SADs at the same time. Additionally, all patients were investigated before starting immunosuppressive therapy; therefore, we can rule out the potential immuno-modifying effects.

First, the distribution of the main populations within the CD45^+^ living cells was determined and compared among the investigated groups. The seven main cell types showed significant differences (**Figure 3**). Among the main immune populations, CD4^−^/CD8^−^ double-negative (DN) T cells, plasmablasts, and CD11c^dim^/CD172a^dim^ cells showed a significantly higher average population percentage in patients with SLE than in all the other groups. No publications are available on the CD11c^dim^/CD172a^dim^ in the context of SLE. In contrast, CD4^+^/CD57^+^ T cells and CD4^+^ NKT cells were present in significantly lower numbers in patients with SLE than in healthy controls and the SSc group. The average population of CD8^+^/CD161^+^/CD28^+^ cytotoxic T cells was significantly higher in healthy individuals than in patients with SADs. Two populations were identified, CD8a^dim^/CD47^dim^ and CD56^dim^/CD98^dim^, with the mean population percentages within CD45^+^ single cells being the lowest in the RA group. In the case of the CD56^dim^/CD98^dim^ population, the difference was significant compared with the SLE and HC groups. This observation is also considered novel.

Second, the expression levels of the 34 markers in the main populations were compared between the groups. In summary, 59 scatter plots showed significant differences between at least two groups (**Supplementary Figures 4**–**12**). The most potent marker is cyclic ADP-ribose hydrolase (CD38). It was highly expressed in a variety of immune cells in all three therapy-naive patient groups compared with HCs: DN T cells, TCRγ/δ^+^ T cells, CD8a^+^ NKT cells, NK cells, and monocytes. Similarly, the CD8a^dim^/CD47^dim^ population in the SLE group showed significantly higher CD38 expression than those in the RA and SSc groups. CD38 expression was also significantly higher in CD8a^+^/CD161^−^ cytotoxic T cells in the SLE group than in the other groups. CD38 as a targeted therapy (daratumumab) has been approved for multiple myeloma, but it has also been suggested for SADs, particularly SLE, where plasma cells do not express CD20, leading to rituximab resistance; however, they highly express the CD38 (**Figure 12A**) (45–47).

Third, each immune cell population was divided into subpopulations (MCs) using the FlowSOM algorithm (**Figures 4**–**13**; **Supplementary Figures 13**–**16**). Subpopulation percentages were compared among the populations in different groups. We identified 121 MCs from 10 major immune cell populations. In addition, T cells were classified into 64 MCs based on the expression of 34 markers. Twenty-three T-cell subpopulations were found with significantly different percentages between at least two groups (**Figures 4**–**8**). Tregs are known to be present at lower frequencies in HCs than in the SSc group (48, 49), and we identified a subpopulation of Tregs (MC03: CD25^+^CD38^−^CD127^−^CD194^+^) within CD4^+^CD57^−^ T cells with a decreased ratio in HCs. Burnst et al. reported elevated expression of CD38 in effector memory CD4^+^ T cells (50). We identified two CD4^+^ T_EM_ which were present in higher percentages in SLE: CD38 negative (MC08: CD45RA^−^CD4^+^CD27^+^CD28^+^CD38^−^CD127^−^CD197^−^) and CD38 positive (MC11: CD45RA^−^CD4^+^CD27^−^CD28^+^CD38^+^CD127^−^CD197^−^), differentiating SLE from therapy-naive RA and SSc. Lima et al. reported that CD38^+^HLA-DR^+^ cytotoxic T cells were elevated in patients with SLE (51). We demonstrated that the MC01 (CD27^−^CD28^−^CD38^+^CD57^+^CD127^−^HLA-DR^+^) population in CD8^+^CD161^−^ T cells was the best in differentiating SLE from RA and SSc. Comte et al. did not observe differences in the ratio of CD8^+^ T_EMRA_ (CD45RA^+^CD197^−^) between HCs and patients with SLE (52). In contrast, we demonstrated that one subpopulation of CD8^+^ T_EMRA_, the MC05 (CD27^−^CD28^−^CD38^−^CD57^+^CD127^+^), was the lowest in SLE compared with HCs, RA, and SSc. Yuan et al. showed a higher percentage of naive CD8^+^ T cells in HCs vs. SLE (53). In addition, the highest percentage of naive CD8^+^ T-cell subpopulation was found in HCs, whereas MC15 (CD27^+^CD28^+^CD45RA^+^CD45RO^−^CD127^+^CD183^+^CD197^+^) was higher in HCs than in SADs. Cho et al. reported that DN MAIT cells were more prevalent in HCs than in patients with RA and SLE (54). Our results also confirmed a lower ratio of MC06 DN T cells (CD38^−^CD127^+^CD161^+^) in patients with SLE. The unequivocal role of TCRγ/δ T cells in the pathogenesis of SADs has been described recently (55, 56). We identified three subpopulations of TCRγ/δ T cells differentiating SLE from HCs, RA, and SSc. Two of these were significantly higher in SLE (MC01: CD27^+^CD197) (MC04: CD45RO^dim^CD45RA^dim^CD57^−^), and one was significantly lower in SLE (MC12: CD45RA^+^CD56^+^CD57^+^).

Subsequently, 57 subpopulations (MCs) of non-T-cell compartments were demonstrated in CD3^−^ cells; among these, 17 populations showed significantly different subpopulation percentages between at least two investigated groups (**Figures 9**–**13**). A lower proportion of CD56^+^ NK cells has been reported in patients with RA and SLE (57, 58). In line with this, we identified one subpopulation of NK cells, MC03 (CD38^−^CD57^−^CD161^−^), with the highest percentage in HCs. Schepis et al. reported an increased frequency of CD56^bright^CD16^−^ NK cells in patients with SLE compared to HCs (59). Based on our study, the number of MC05 (CD56^bright^CD45RA^−^CD16^low^) cells was the highest in SLE. In contrast to MC05, the MC10 (CD8a^+^CD38^+^CD57^+^) NK cell population was the lowest in patients with SLE. A lower ratio of CD56^+^CD57^+^ NK cells in SLE compared to HCs was reported previously by Lu et al. (60); however, our data also included comparisons with RA and SSc. We identified a subpopulation of CD56^dim^/CD98^dim^ MC05 (CD16^+^/CD57^+^/CD183^−^) cells with a significant decrease in SLE, but there is a lack of data on these cells in the context of SADs. Amu et al. showed that CD25^+^CD20^+^CD27^+^ B cells were the lowest in patients with SLE compared with HCs (61). Our results also supported the lowest percentage of MC08 (CD25^+^CD20^+^CD27^+^IgD^+^) B cells in SLE compared with that in HCs, RA, and SSc. Rincon-Arevalo et al. reported an increased proportion of CD11c^+^ B cells in patients with SLE (62). Additionally, we differentiated two CD11c subsets of B cells with the highest frequency in SLE: MC09 (CD11c^+^CD38^−^CD185^−^) and MC16 (CD11c^+^CD183^+^). B cells expressing CD11c and lacking CD21 expression (age-associated B cells = ABCs) are reported as an increasing population in SLE (63). Indeed, our MC16 population expresses CD11c, but we highlighted the co-expression of CD183 (or panel missed CD21), which differentiates it from the ABCs. The M16 B-cell population (IgD^−^CD27^+^) is different also from the double-negative IGD^−^CD27^−^ population that was described by Wang et al. in SLE (64). The peripheral composition of plasmablasts was shared with CD27^−^ and CD27^+^ MCs with the highest and lowest frequencies in SLE, respectively. Toapanta et al. reported the induction of CD27 plasmablasts after Shigella LPS treatment, with a correlation between IgA and IgG production (65). However, limited data are available on CD27^−^ plasmablasts in SLE. Lesco et al. showed that CD14^bright^CD16^−^ classic monocytes were increased in SSc patients compared with HCs (66). A subpopulation of classic monocytes was identified by our research group, MC10 (CD16^−^CD25^+^CD127^−^HLA-DR^−^), with elevated levels in all investigated SADs compared with HCs. Additionally, we found two intermediate (CD14^bright^/CD16^+^) monocyte populations, MC11 (CD16^+^CD25^+^CD183^−^) and MC12 (CD16^+^CD25^+^CD183^+^), which were higher in SSc than in HCs, RA, and SLE.

In summary, we highlight seven metaclusters, each differentiating one group from the other three. In HCs, compared with patients with SADs, the following subpopulations showed significantly lower subpopulation percentages: MC08 (CD27^+^/CD28^+^/CD38^−^CD127^−^/CD197^dim^) in CD4^+^/CD57^−^ T cells, and the SLE group also differed from the other two SAD groups. The subpopulations MC04 (CD45RO^dim^/CD45RA^dim^/CD57^−^) of TCRγ/δ^+^ T cells and MC10 (CD16^−^/CD25^+^/CD127^−^/HLA-DR^−^) of monocytes had the lowest percentage in HCs. In contrast, the following subpopulation percentages were significantly higher in HCs than in SADs: MC06 (CD27^+^/CD28^+^/CD38^−^CD57^+^/CD127^−^) in CD8a^+^/CD161^−^ T cells and MC03 (CD38^−^/CD57^−^/CD161) in NK cells. In patients with SLE, we detected a significantly higher subpopulation percentages of MC07 (CD38^+^/CD196^−^/IgD^−^) in B cells and MC01 (CD27^−^) in plasmablasts compared with the other three groups. The findings of our study showed that the peripheral immune landscape demonstrated circulating immune cell attributes that discriminated the three SADs, therapy-naive RA, SSc, and SLE, from each other, as well as from HCs.

This study is based on several years of patient sample collection. For all inflammatory rheumatic diseases, the time between the initial symptoms and the actual diagnosis can be years. In addition, patients are almost invariably admitted to specialist care centers following a certain form of anti-inflammatory or immunosuppressive treatment. Both the prolonged duration of illness and the treatments used can significantly alter the patient’s immunophenotype. Mapping the immunophenotype of early and untreated patients can be of importance in several ways. In the early stages of the disease, there can be a lot of overlap between different syndromes. In many cases, they are identified as an undifferentiated autoimmune syndrome. Early mapping of the immunophenotype can help in early diagnosis. Knowledge of the immunophenotype prior to therapy can also be a prognostic marker for subsequent response to therapy. Changes in disease activity can be used to identify markers of disease severity. This may provide the basis for further prospective analysis following the current study. Identifying difficult-to-treat patient groups is another major clinical challenge. Furthermore, the results of our present study may help to map this patient group, including various possible organ-specific immunological processes. Our results, including significant differences in several main cell populations, marker expression intensities, and metaclusters, may contribute to clarify the prior described, challenging autoimmune diseases. Additionally, our dataset about early, untreated patients may show overt disease pathology related to the etiology of the disease unveiling potential therapeutic targets that could contribute to the development of novel therapies.

## Data availability statement

The raw data supporting the conclusions of this article will be made available by the authors, without undue reservation.

## Ethics statement

Details about the study design and handling of biological materials were submitted to the Human Investigation Review Board of the University of Szeged under the 149/2019-SZTE Project Identification code. The studies were conducted in accordance with the local legislation and institutional requirements. The participants provided their written informed consent to participate in this study.

## Author contributions

JB: Conceptualization, Data curation, Formal Analysis, Investigation, Methodology, Software, Validation, Visualization, Writing – original draft. ÁZ: Investigation, Methodology, Writing – original draft. VB: Investigation, Writing – original draft. LP: Conceptualization, Funding acquisition, Project administration, Resources, Writing – original draft. AB: Conceptualization, Funding acquisition, Investigation, Resources, Supervision, Writing – original draft, Writing – review & editing. GS: Conceptualization, Funding acquisition, Project administration, Resources, Supervision, Visualization, Writing – original draft, Writing – review & editing.

## Supplementary material

Supplementary Figure 1 Determination of live single cells and debarcoding of the CyTOF FCS files. Cells were gated first negative for 140Ce and 142Ce calibration bead specific metal tags (upper row on the left). Singlets were gated based on 191Ir DNA labeling (upper row in the middle). Live cells were gated based on negativity for 103Rh cationic nucleic acid intercalator Live/Dead reagent (Fluidigm) (upper row on the right). Debarcoding of the patients was carried out using the gating on CD45+ positive cells such as the following: HC: 116Cd CD45+, RA: 89Y CD45+, SSc: 114Cd CD45+, SLE: 106Cd CD45+.

Supplementary Figure 2 Data clarification of CyTOF FCS files based on manual gating excluding cell doublets. Double negative cells were processed further during the data analysis for CD56-/CD294-, CD3-/CD14-, CD19-/CD14-, CD19-/CD3-, CD56-/CD19-, CD20-/CD16-.

Supplementary Figure 3 The percentage of the main immune subsets within the matured living peripheral CD45+ leukocytes. The non-significant differences are demonstrated here, significant differences are demonstrated in **Figures 2**. The values shown on the column bar are from the bottom to the top: lower bar = minimum value, bottom line of the chart = lower quartile (Q1), middle line = median, top line of the chart = upper quartile (Q3), upper bar = maximum value.

Supplementary Figure 4 The tSNE plots of the expression profile of 34 markers. The areas of the tSNE plots corresponds to the main 17 immune subset described in **Figures 1A**. The red coloration is proportional with the higher, the blue coloration is proportional with the lower expression levels within the 17 immune subsets. Aggregated data of the 52 cases.

Supplementary Figure 5 The significant differences in the marker expression profile (median metal intensity) of CD4+/CD57- T-cells (upper row) and the CD8a+/CD161- T-cells (lower row). The statistical method (WA = Welch-ANOVA, or ANOVA = one way ANOVA) is shown on the top of each column bar. Significance was accepted when the q value of the false discovery rate (FDR) was below 0.1 and *p<0.05, **p<0.01. The values shown on the column bar are from the bottom to the top: lower bar = minimum value, bottom line of the chart = lower quartile (Q1), middle line = median, top line of the chart = upper quartile (Q3), upper bar = maximum value.

Supplementary Figure 6 The significant differences in the marker expression profile (median metal intensity) of CD8+/CD161+/CD28+ T-cells (upper row) and the CD8a+dim T-cells (lower row). The statistical method (KW= Kruskal Wallis test; WA = Welch-ANOVA, or ANOVA = one way ANOVA) is shown on the top of each column bar. Significance was accepted when the q value of the false discovery rate (FDR) was below 0.1 and *p<0.05, **p<0.01, ***p<0.001, ****p<0.0001. The values shown on the column bar are from the bottom to the top: lower bar = minimum value, bottom line of the chart = lower quartile (Q1), middle line = median, top line of the chart = upper quartile (Q3), upper bar = maximum value.

Supplementary Figure 7 The significant differences in the marker expression profile (median metal intensity) of CD3+/CD4-/CD8a-T-cells. The statistical method (KW= Kruskal Wallis test; WA = Welch-ANOVA, or ANOVA = one way ANOVA) is shown on the top of each column bar. Significance was accepted when the q value of the false discovery rate (FDR) was below 0.1 and *p<0.05, **p<0.01, ***p<0.001, ****p<0.0001. The values shown on the column bar are from the bottom to the top: lower bar = minimum value, bottom line of the chart = lower quartile (Q1), middle line = median, top line of the chart = upper quartile (Q3), upper bar = maximum value.

Supplementary Figure 8 The significant differences in the marker expression profile (median metal intensity) of TCRgd+ T-cells (upper row), CD4+ NKT, or CD8a+ NKT cells (lower row). The statistical method (KW= Kruskal Wallis test; WA = Welch-ANOVA, or ANOVA = one way ANOVA) is shown on the top of each column bar. Significance was accepted when the q value of the false discovery rate (FDR) was below 0.1 and *p<0.05, **p<0.01, ***p<0.001, ****p<0.0001. The values shown on the column bar are from the bottom to the top: lower bar = minimum value, bottom line of the chart = lower quartile (Q1), middle line = median, top line of the chart = upper quartile (Q3), upper bar = maximum value.

Supplementary Figure 9 The significant differences in the marker expression profile (median metal intensity) of NK cells (upper row), CD56dim/CD98 dim cells (lower row). The statistical method (KW= Kruskal Wallis test; WA = Welch-ANOVA, or ANOVA = one way ANOVA) is shown on the top of each column bar. Significance was accepted when the q value of the false discovery rate (FDR) was below 0.1 and *p<0.05, **p<0.01, ***p<0.001, ****p<0.0001. The values shown on the column bar are from the bottom to the top: lower bar = minimum value, bottom line of the chart = lower quartile (Q1), middle line = median, top line of the chart = upper quartile (Q3), upper bar = maximum value.

Supplementary Figure 10 The significant differences in the marker expression profile (median metal intensity) of B-cells (upper and middle rows), plasmablasts (lower row). The statistical method (KW= Kruskal Wallis test; ANOVA = one way ANOVA) is shown on the top of each column bar. Significance was accepted when the q value of the false discovery rate (FDR) was below 0.1 and *p<0.05, **p<0.01, ***p<0.001, ****p<0.0001. The values shown on the column bar are from the bottom to the top: lower bar = minimum value, bottom line of the chart = lower quartile (Q1), middle line = median, top line of the chart = upper quartile (Q3), upper bar = maximum value.

Supplementary Figure 11 he significant differences in the marker expression profile (median metal intensity) of Monocytes (upper row), CD11cdim/ CD172adim cells (middle and lower rows). The statistical method (KW= Kruskal Wallis test; WA = Welch-ANOVA, or ANOVA = one way ANOVA) is shown on the top of each column bar. Significance was accepted when the q value of the false discovery rate (FDR) was below 0.1 and *p<0.05, **p<0.01, ***p<0.001, ****p<0.0001. The values shown on the column bar are from the bottom to the top: lower bar = minimum value, bottom line of the chart = lower quartile (Q1), middle line = median, top line of the chart = upper quartile (Q3), upper bar = maximum value.

Supplementary Figure 12 The significant differences in the marker expression profile (median metal intensity) of mDCs cells (upper row), pDCs cells (lower row). The statistical method (KW= Kruskal Wallis test; WA = Welch-ANOVA, or ANOVA = one way ANOVA) is shown on the top of each column bar. Significance was accepted when the q value of the false discovery rate (FDR) was below 0.1 and *p<0.05, **p<0.01, ***p<0.001. The values shown on the column bar are from the bottom to the top: lower bar = minimum value, bottom line of the chart = lower quartile (Q1), middle line = median, top line of the chart = upper quartile (Q3), upper bar = maximum value.

Supplementary Figure 13 Visualizations, tSNE maps of the subpopulations (metaclusters, MCs) defined by the FlowSOM analysis. **(A)** The 2–dimensional viSNE plots of the 20 MCs of CD4+/CD57- T-cells (left). **(B)** The 2–dimensional viSNE plots of the 16 MCs of CD8+/CD161- T-cells (left). **(C)** The 2–dimensional viSNE plots of the 10 MCs of CD8adim/CD47dim T-cells (left). One dot corresponds to one cell (left). The different colors represent the different MCs (left). The red arrows highlight the significant differences in the distribution of MCs among the studied groups. The cell density plots demonstrate the number of cells in the MCs within the studied groups (right). Red color is proportional with high, blue color is proportional with low cell density (right). Red boxes and Arabic numbers demonstrate the distribution of MCs with significant differences among the studied groups.

Supplementary Figure 14 Visualisations, viSNE maps of the subpopulations (metaclusters, MCs) defined by the FlowSOM analysis. **(A)** The 2–dimensional viSNE plots of the 6 MCs of CD4-/CD8- T-cells (left). **(B)** The 2–dimensional viSNE plots of the 12 MCs of TCRgd+T-cells (left). **(C)** The 2–dimensional viSNE plots of the 14 MCs of NK cells (left). One dot corresponds to one cell (left). The different colors represent the different MCs (left). The red arrows highlight the significant differences in the distribution of MCs among the studied groups. The cell density plots demonstrate the number of cells in the MCs within the studied groups (right). Red color is proportional with high, blue color is proportional with low cell density (right). Red boxes and Arabic numbers demonstrate the distribution of MCs with significant differences among the studied groups.

Supplementary Figure 15 Visualisations, viSNE maps of the subpopulations (metaclusters, MCs) defined by the FlowSOM analysis. **(A)** The 2–dimensional viSNE plots of the 7 MCs of CD56dim/CD98dim cells (left). **(B)** The 2–dimensional viSNE plots of the 19 MCs of B-cells (left). **(C)** The 2–dimensional viSNE plots of the 2 MCs of plasmablasts (left). One dot corresponds to one cell (left). The different colors represent the different MCs (left). The red arrows highlight the significant differences in the distribution of MCs among the studied groups. The cell density plots demonstrate the number of cells in the MCs within the studied groups (right). Red color is proportional with high, blue color is proportional with low cell density (right). Red boxes and Arabic numbers demonstrate the distribution of MCs with significant differences among the studied groups.

Supplementary Figure 16 Visualisations, viSNE maps of the subpopulations (metaclusters, MCs) defined by the FlowSOM analysis. The 2–dimensional viSNE plots of the 15 MCs of Monocytes (left). One dot corresponds to one cell (left). The different colors represent the different MCs (left). The red arrows highlight the significant differences in the distribution of MCs among the studied groups. The cell density plots demonstrate the number of cells in the MCs within the studied groups (right). Red color is proportional with high, blue color is proportional with low cell density (right). Red boxes and Arabic numbers demonstrate the distribution of MCs with significant differences among the studied groups.
