# Supplementary material for: Comparative single-cell multiplex immunophenotyping of therapy-naive patients with rheumatoid arthritis, systemic sclerosis, and systemic lupus erythematosus shed light on disease-specific composition of the peripheral immune system
Source: Front Immunol. 2024 Apr 25;15:1376933. doi: 10.3389/fimmu.2024.1376933 (PMC11079270; doi:10.3389/fimmu.2024.1376933)
Supplement: Supplementary Figure 1 — Determination of live single cells and debarcoding of the CyTOF FCS files. Cells were gated first negative for 140Ce and 142Ce calibration bead specific metal tags (upper row on the left). Singlets were gated based on 191Ir DNA labeling (upper row in the middle). Live cells were gated based on negativity for 103Rh cationic nucleic acid intercalator Live/Dead reagent (Fluidigm) (upper row on the right). Debarcoding of the patients was carried out using the gating on CD45+ positive cells such as the following: HC: 116Cd CD45+, RA: 89Y CD45+, SSc: 114Cd CD45+, SLE: 106Cd CD45+. [file DataSheet_1.pdf]

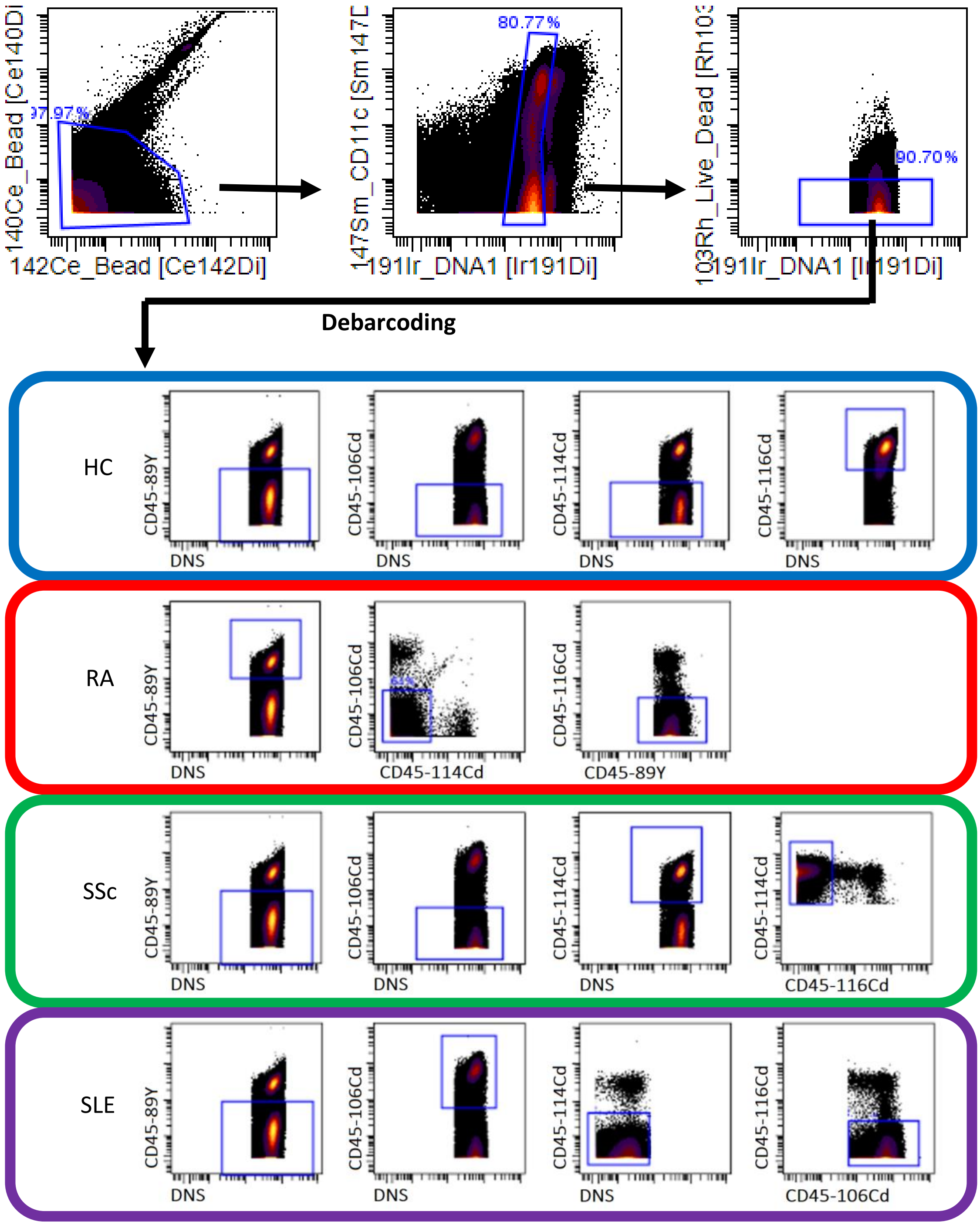

Supplementary Figure 1.

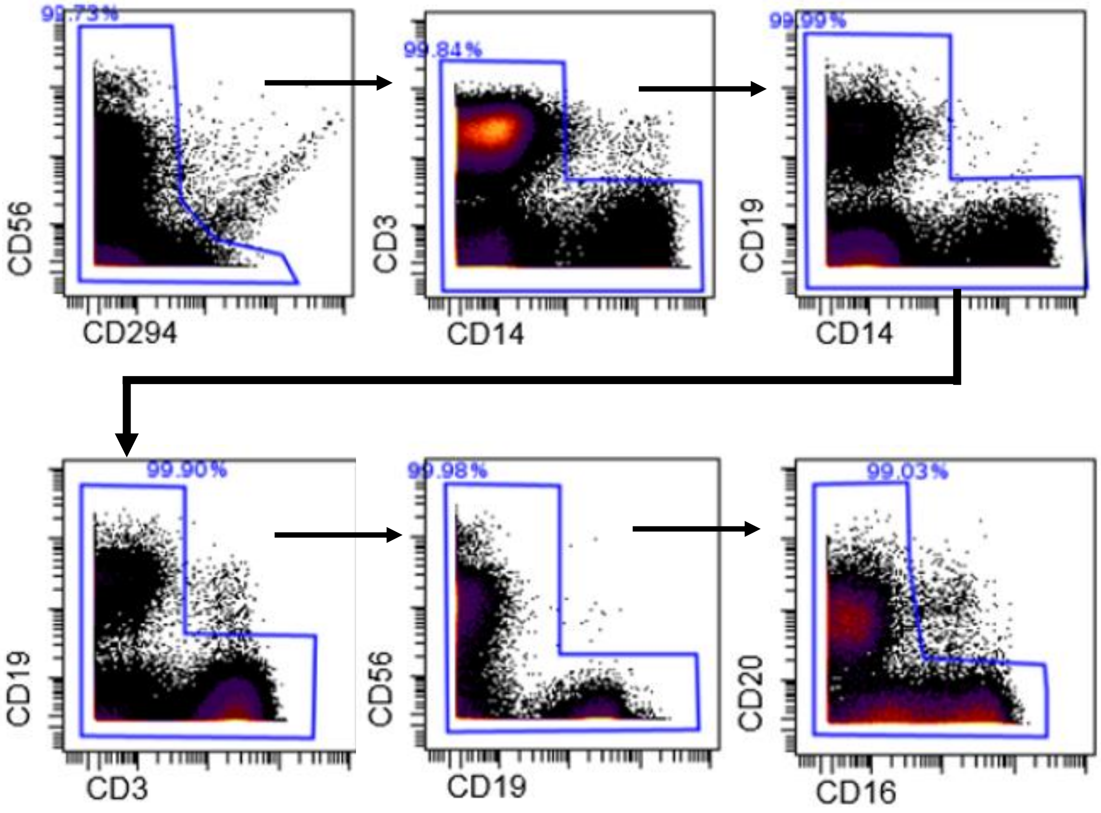

Supplementary Figure 2.

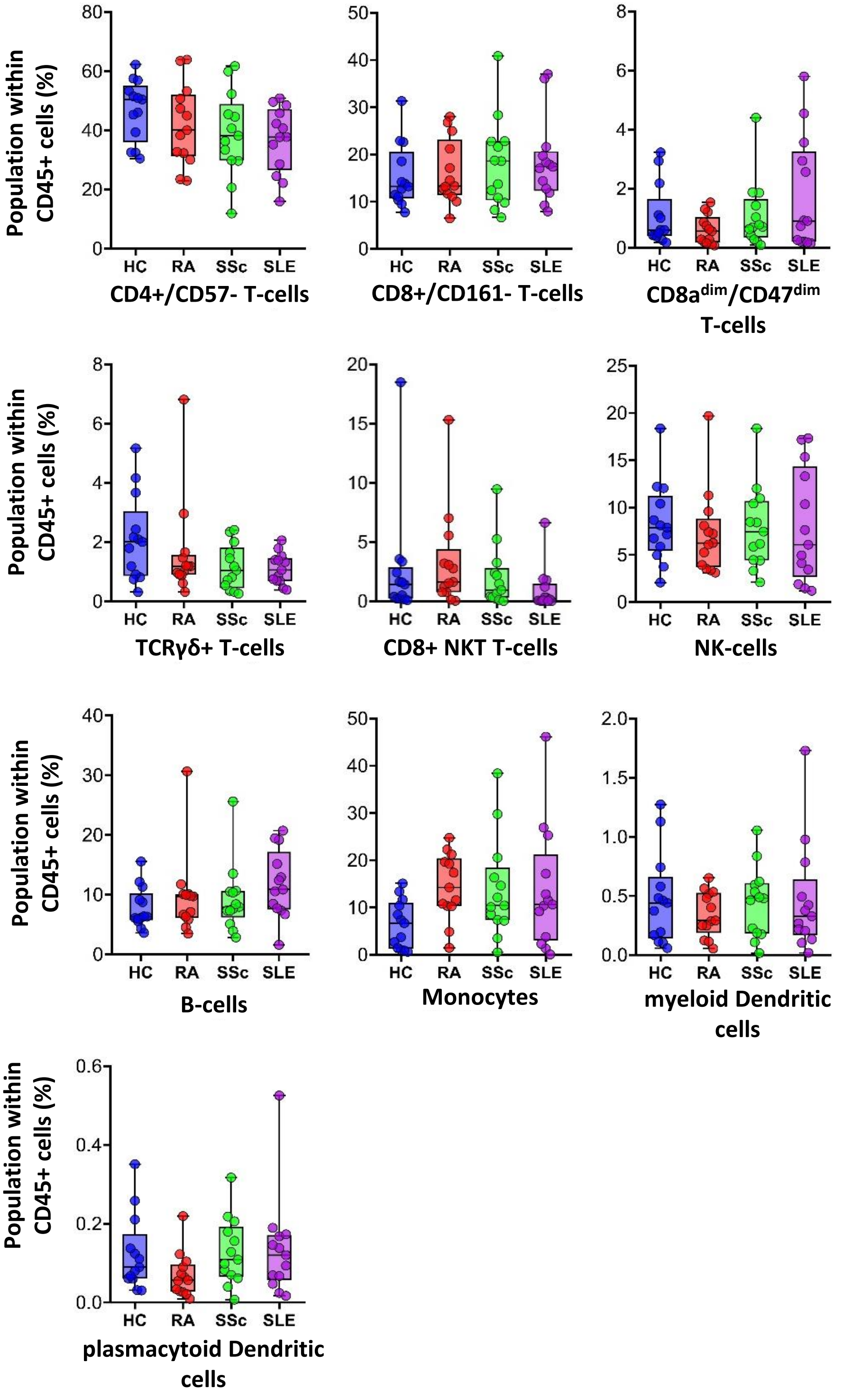

Supplementary Figure 3.

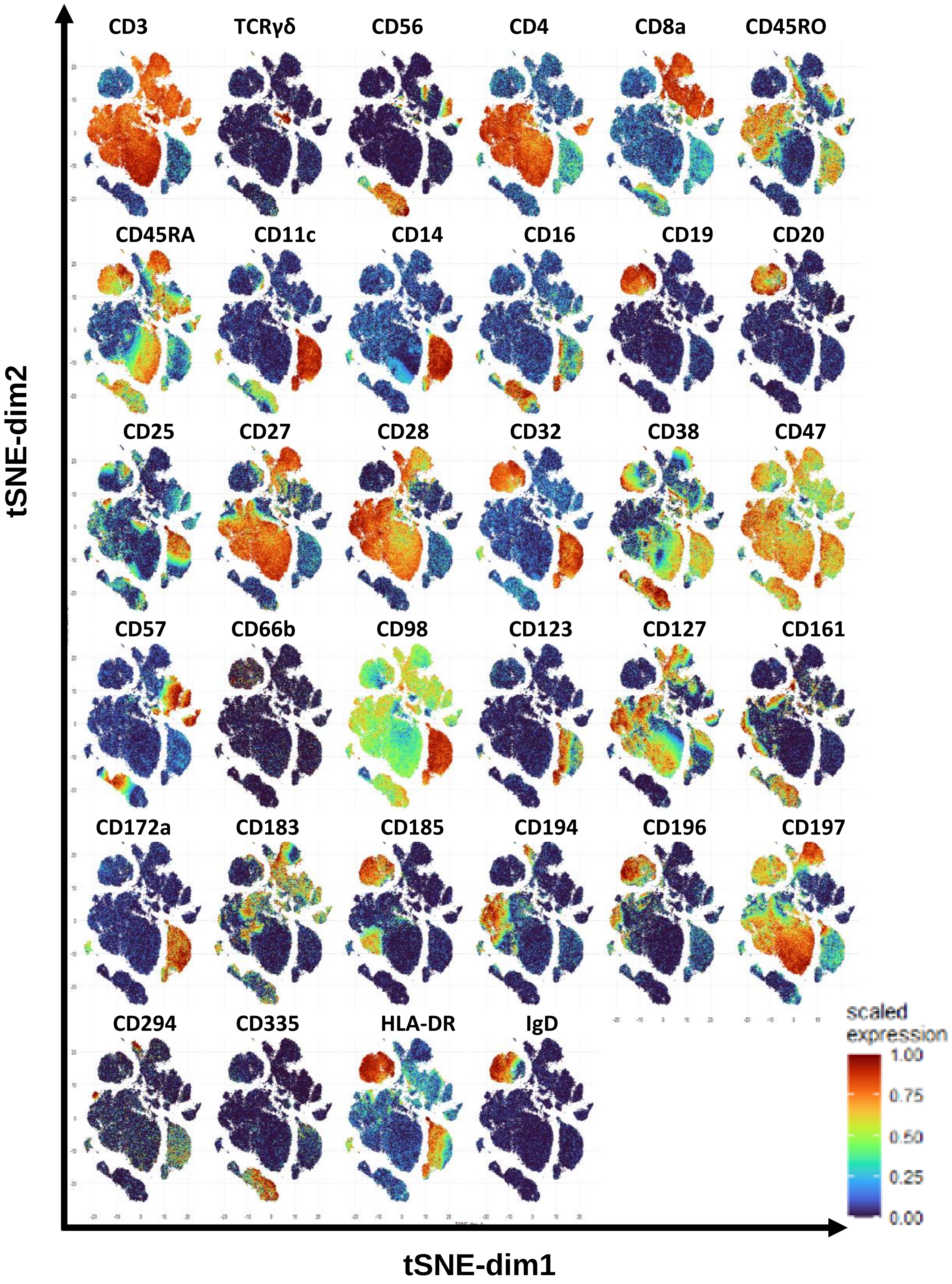

Supplementary Figure 4.

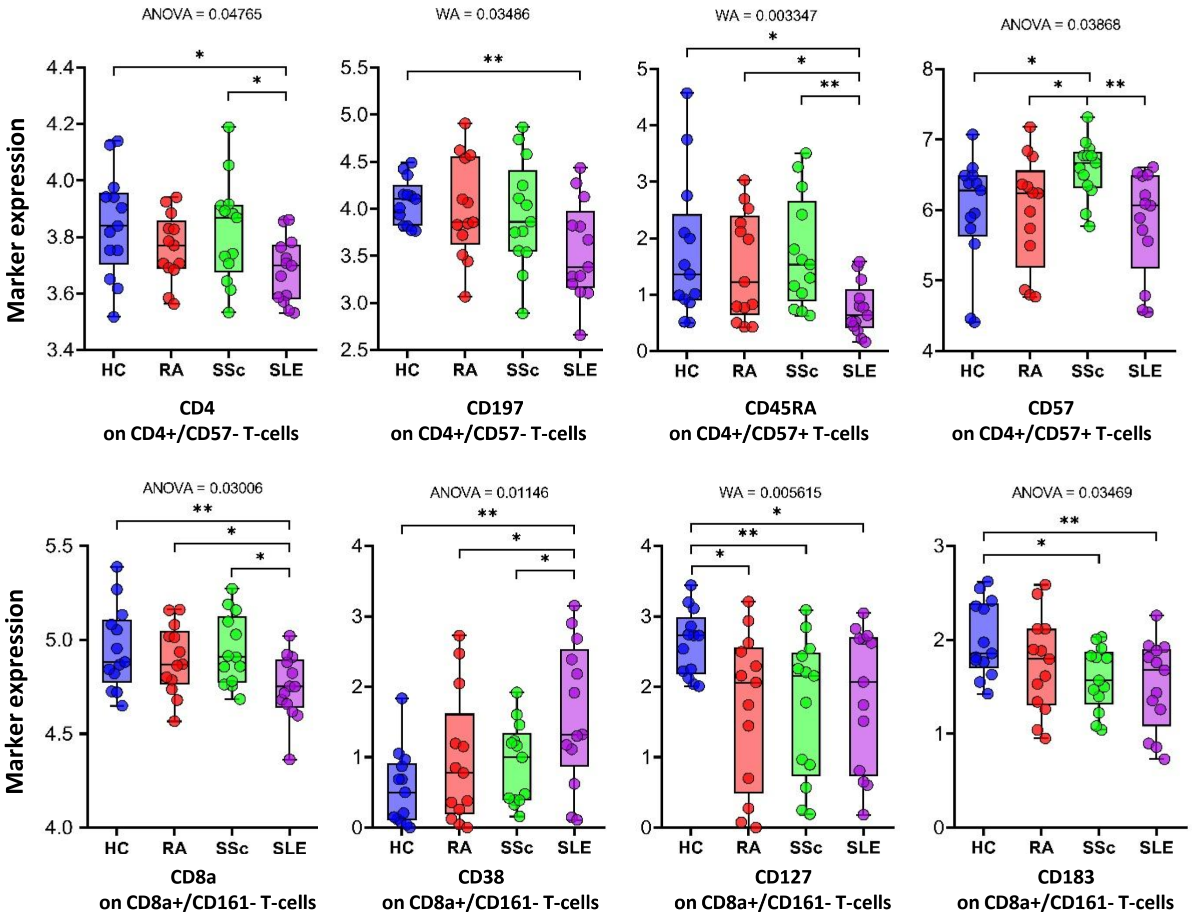

Supplementary Figure 5.

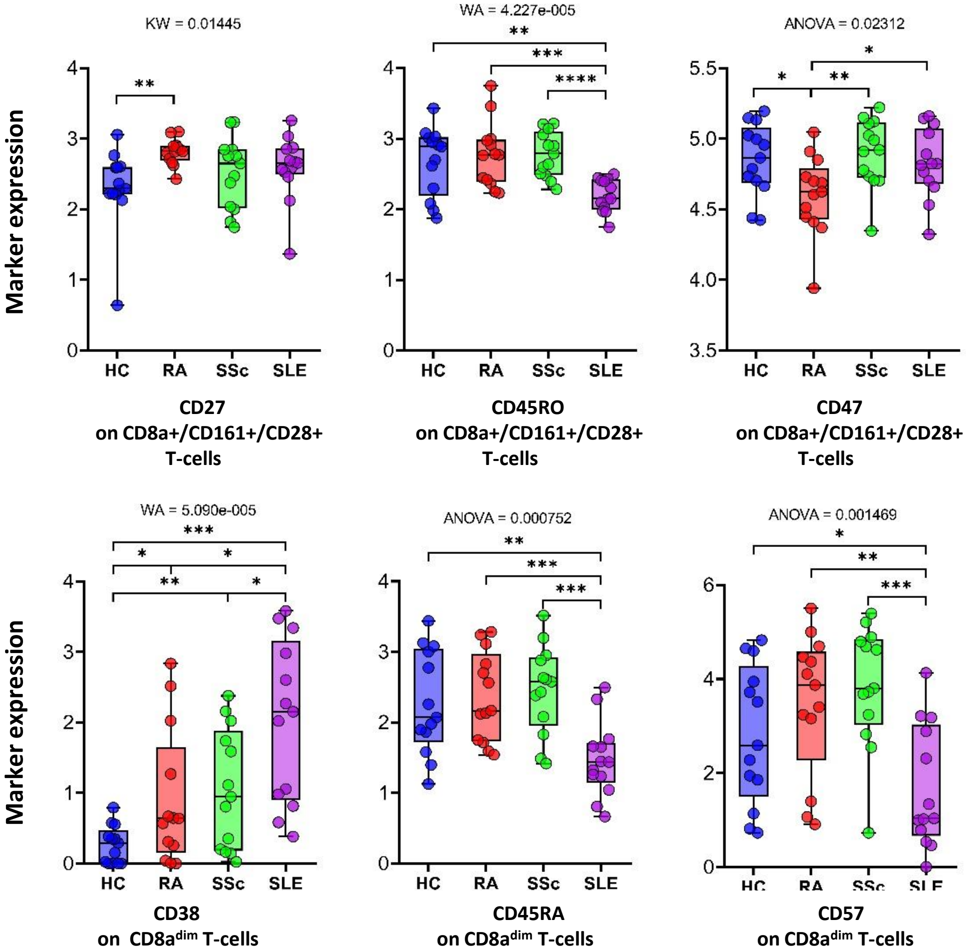

Supplementary Figure 6.

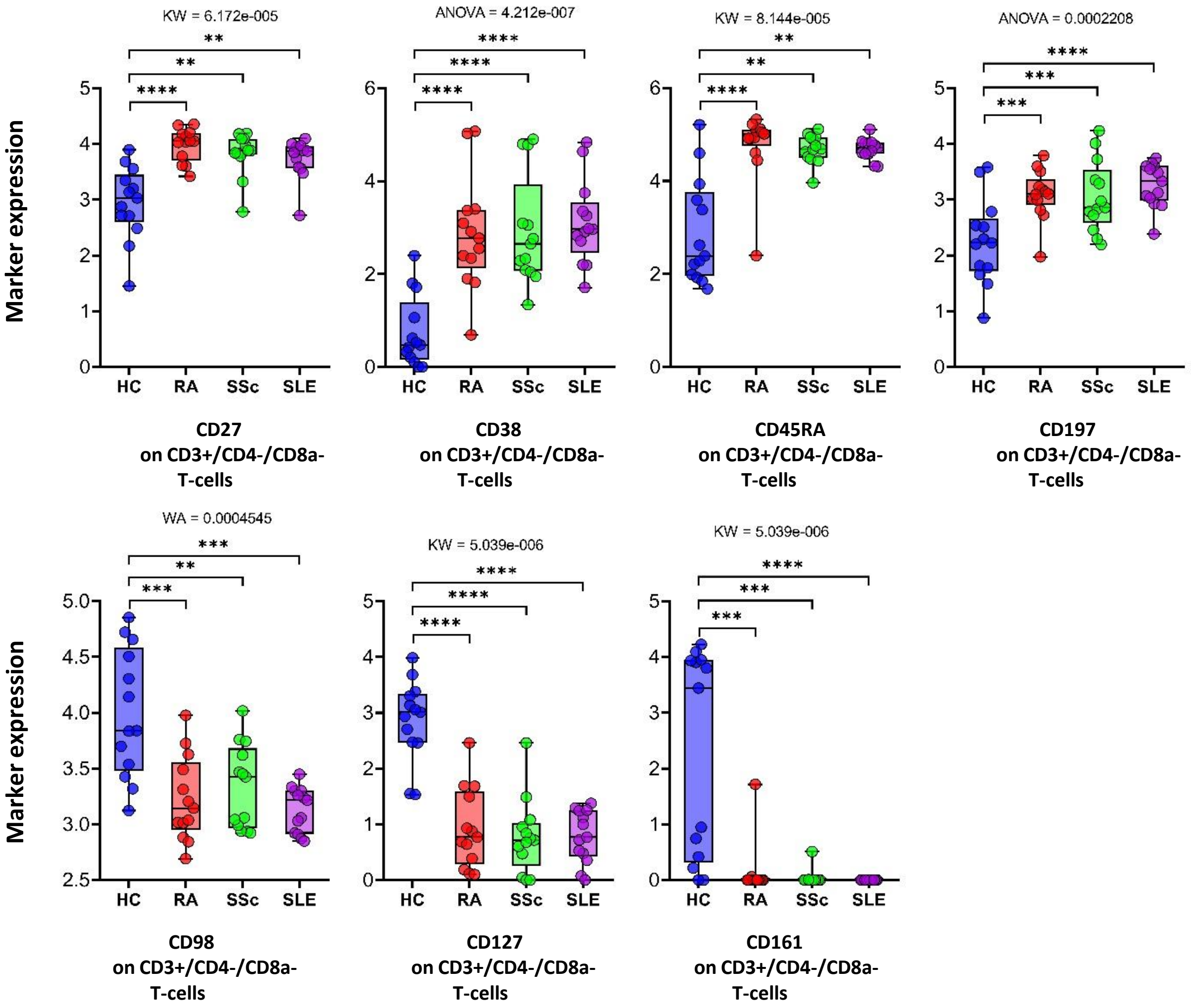

Supplementary Figure 7.

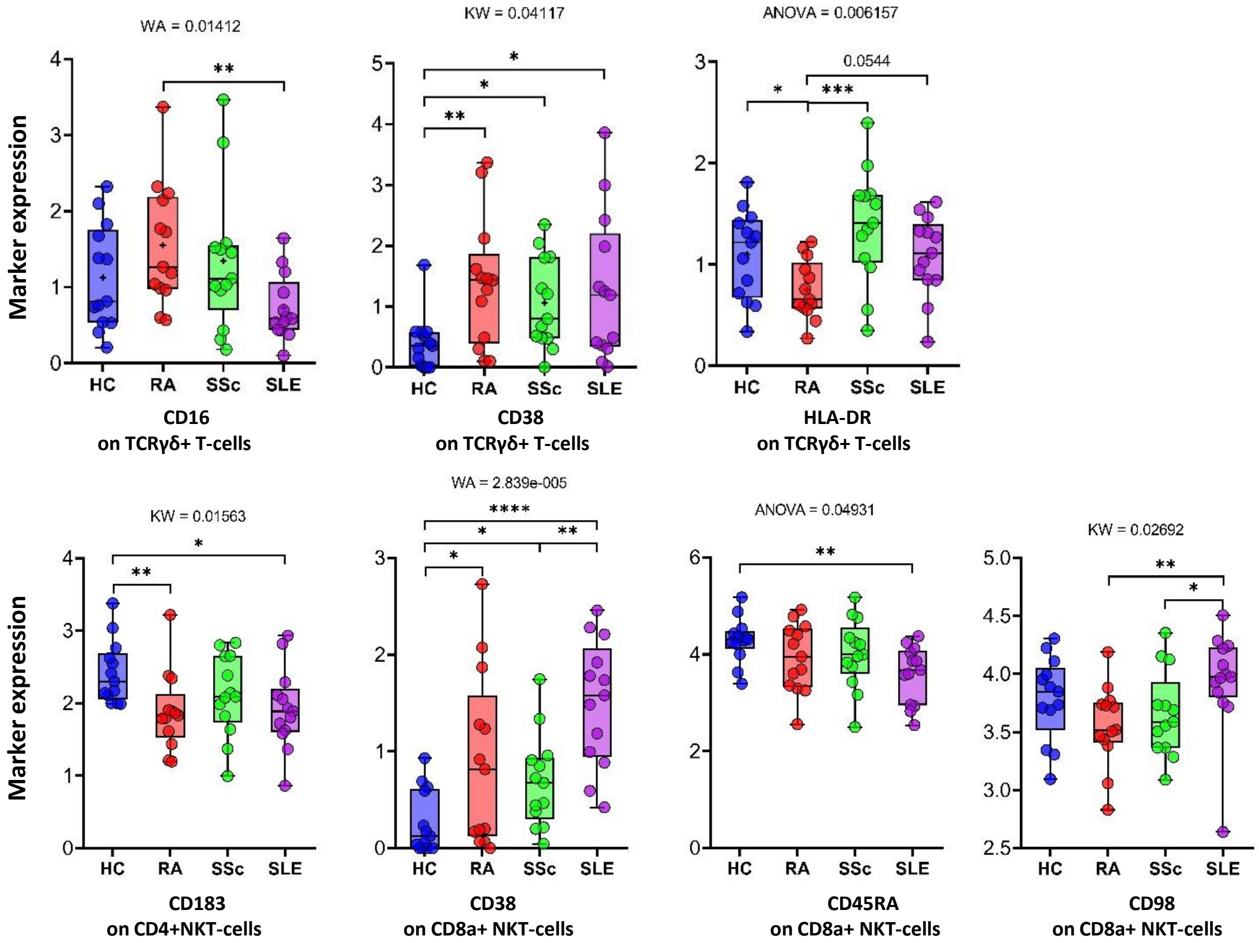

Supplementary Figure 8.

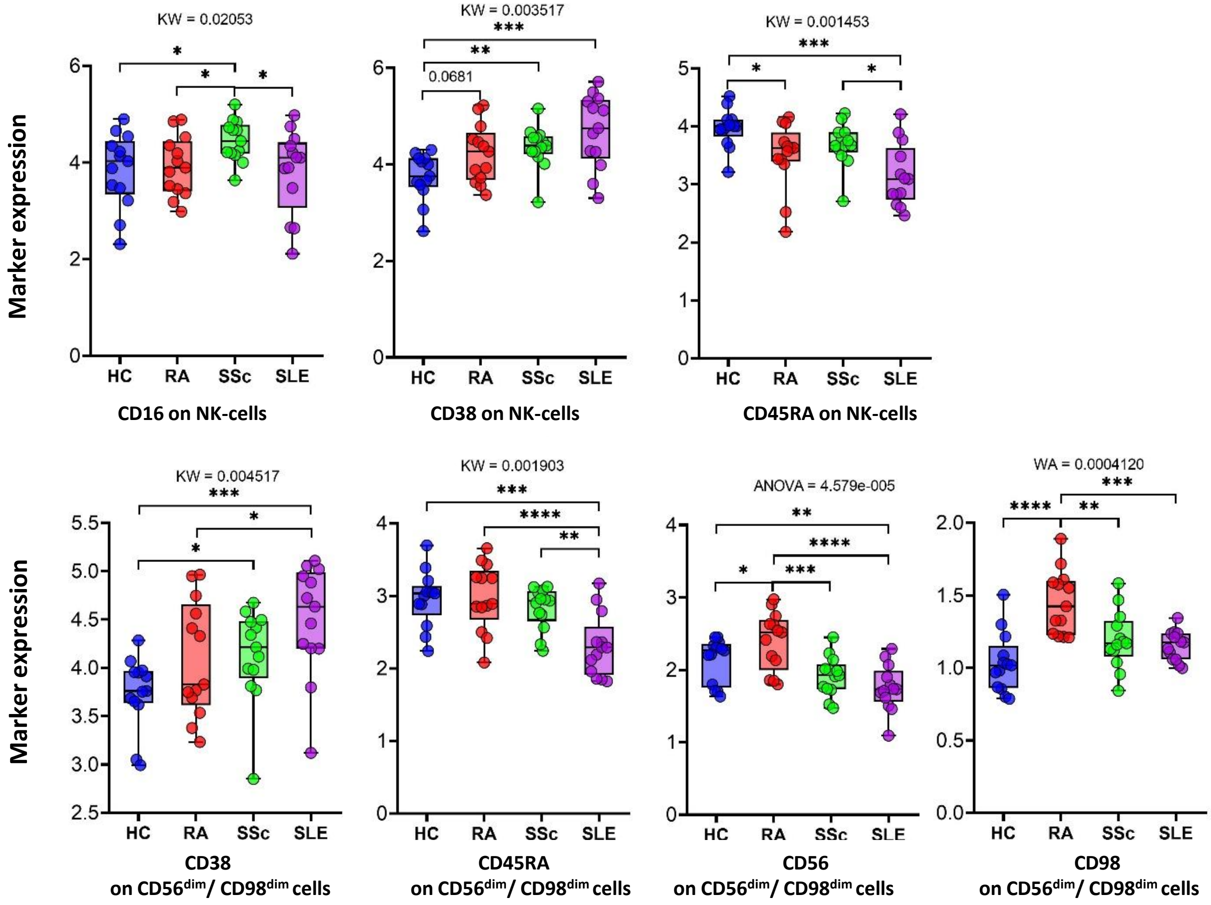

Supplementary Figure 9.

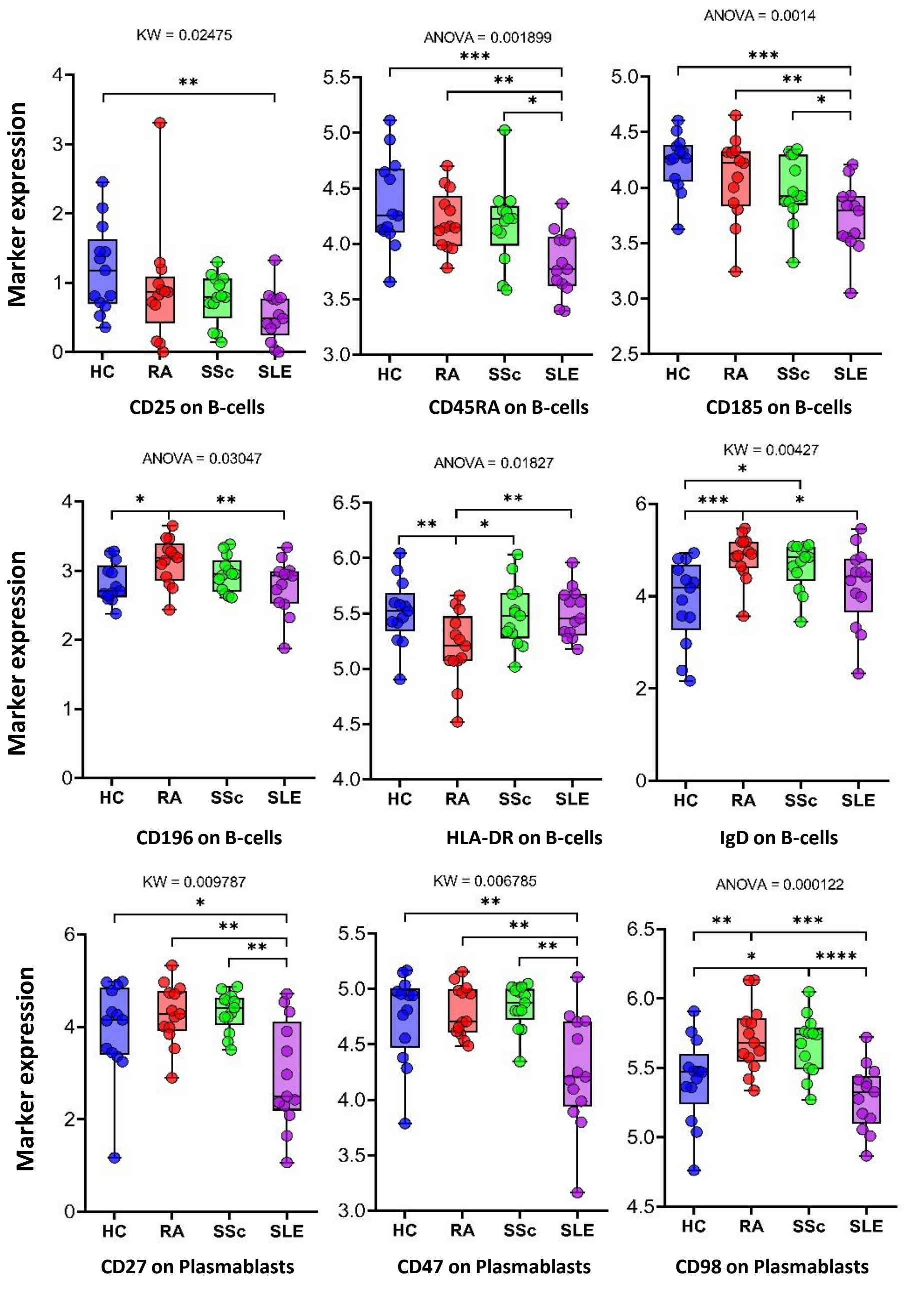

Supplementary Figure 10.

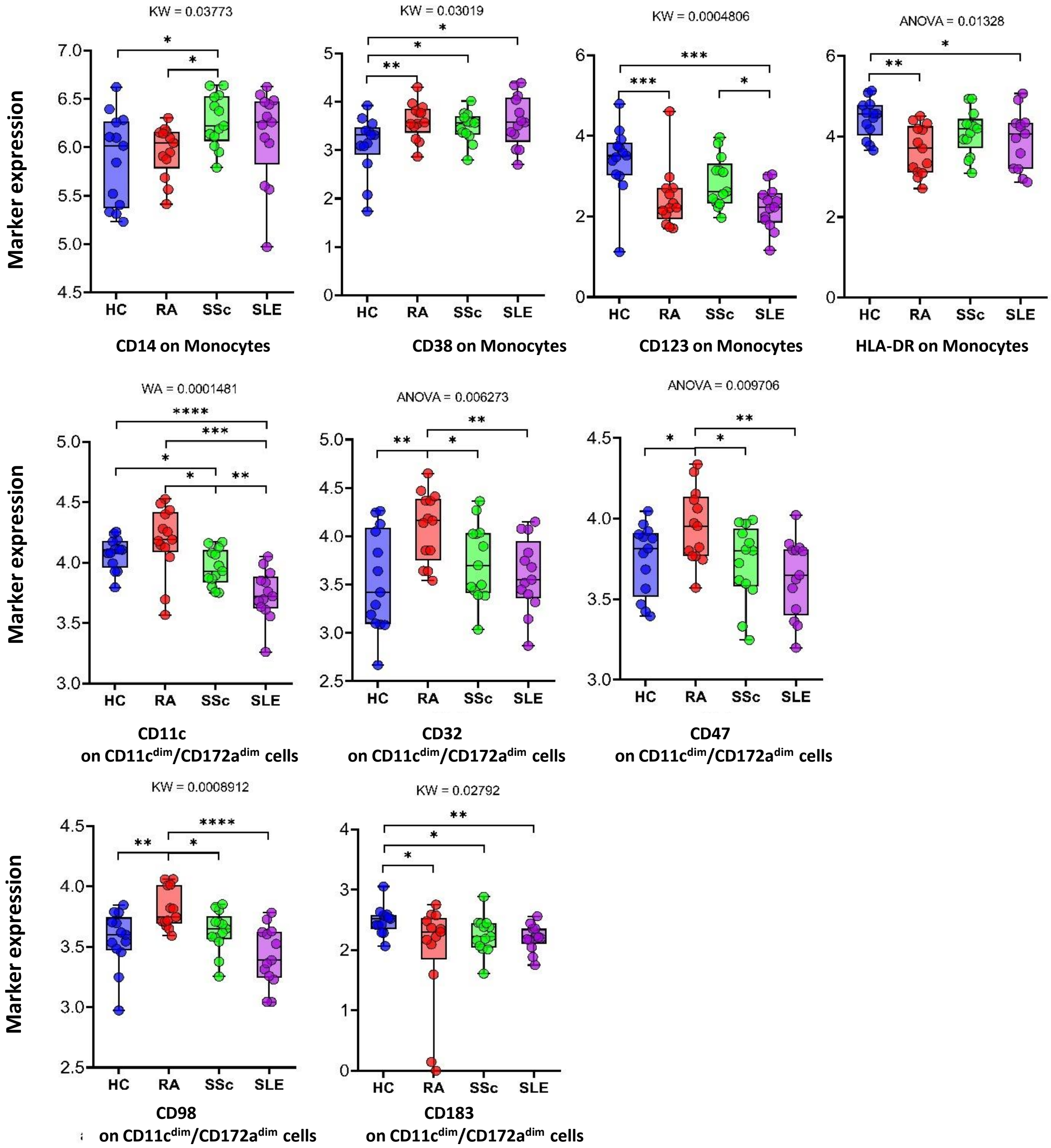

Supplementary Figure 11.

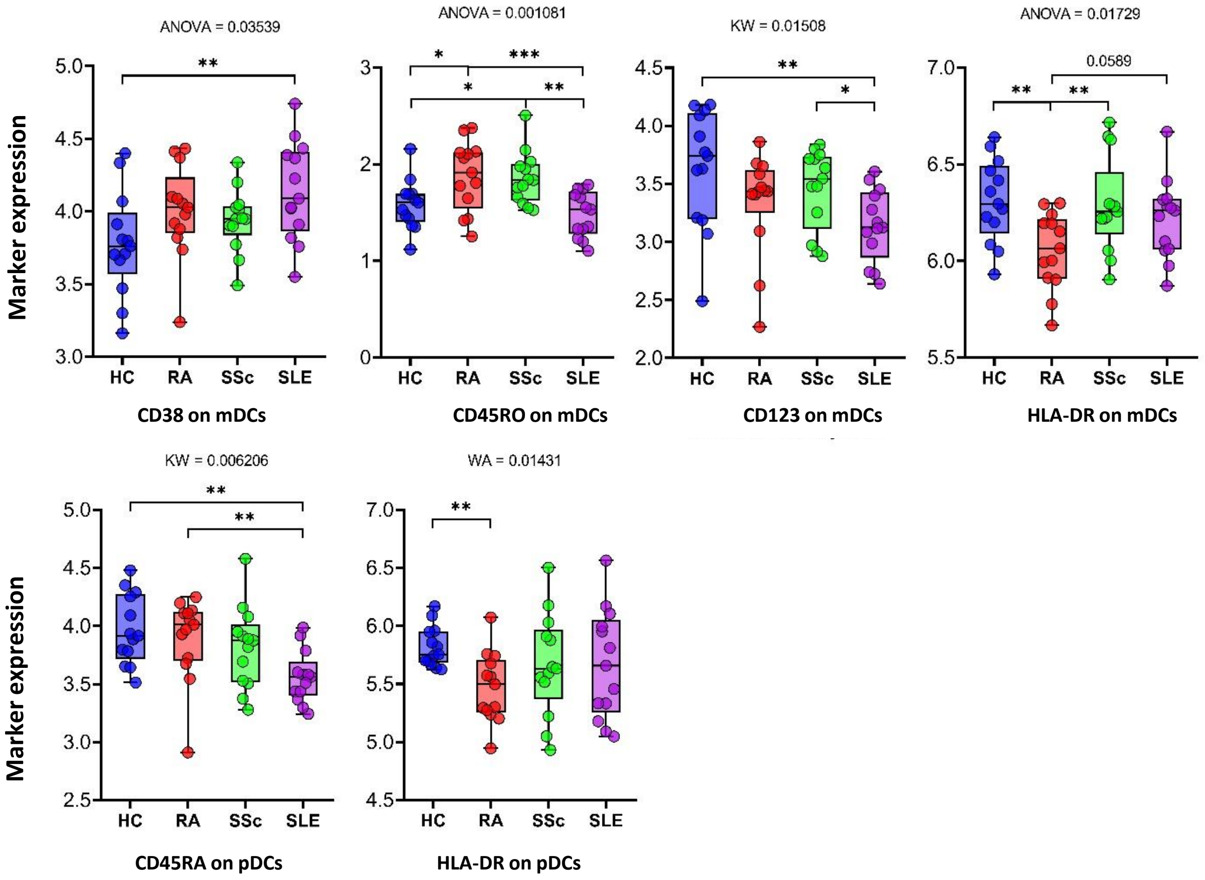

Supplementary Figure 12.

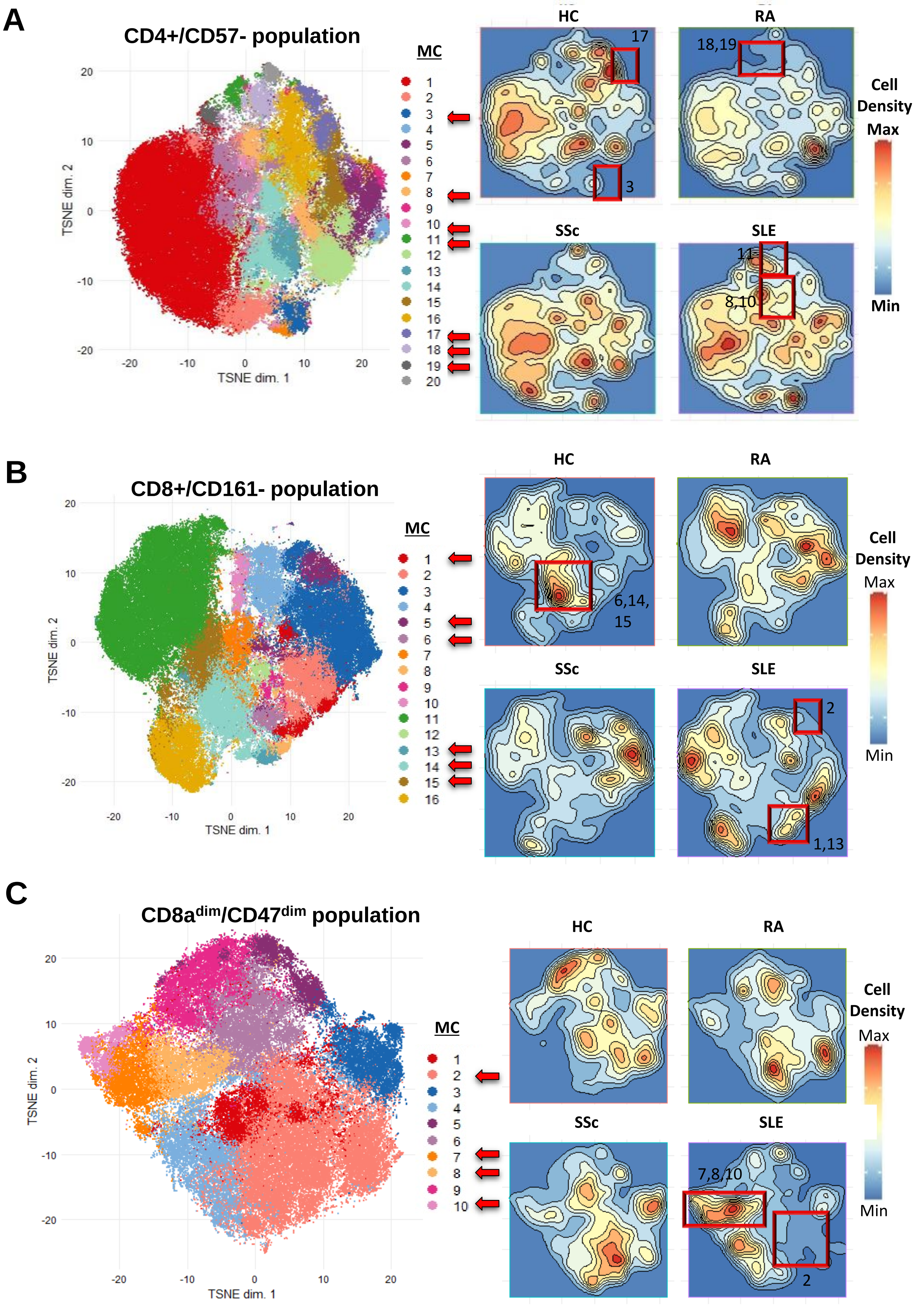

Supplementary Figure 13.

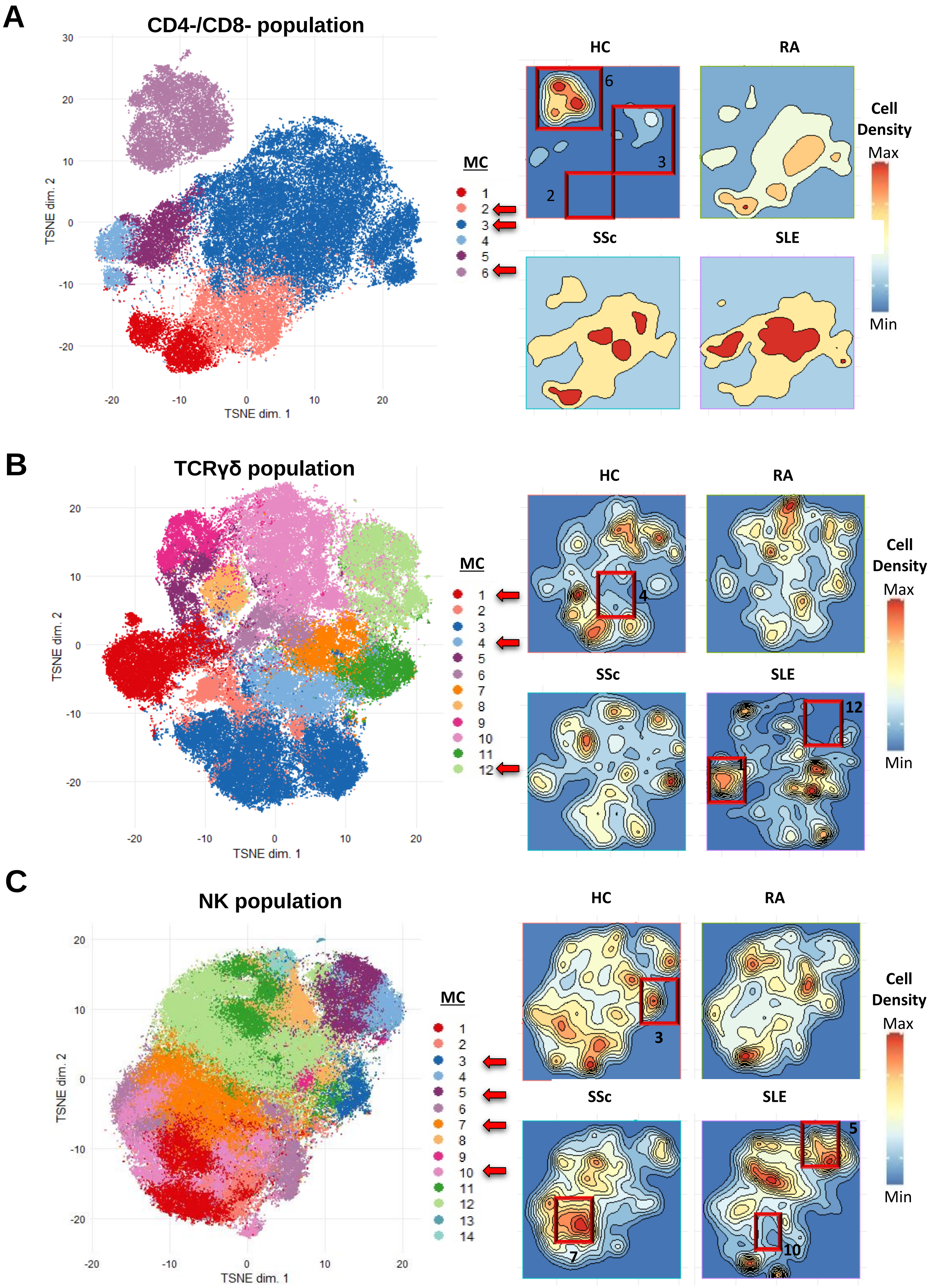

Supplementary Figure 14.

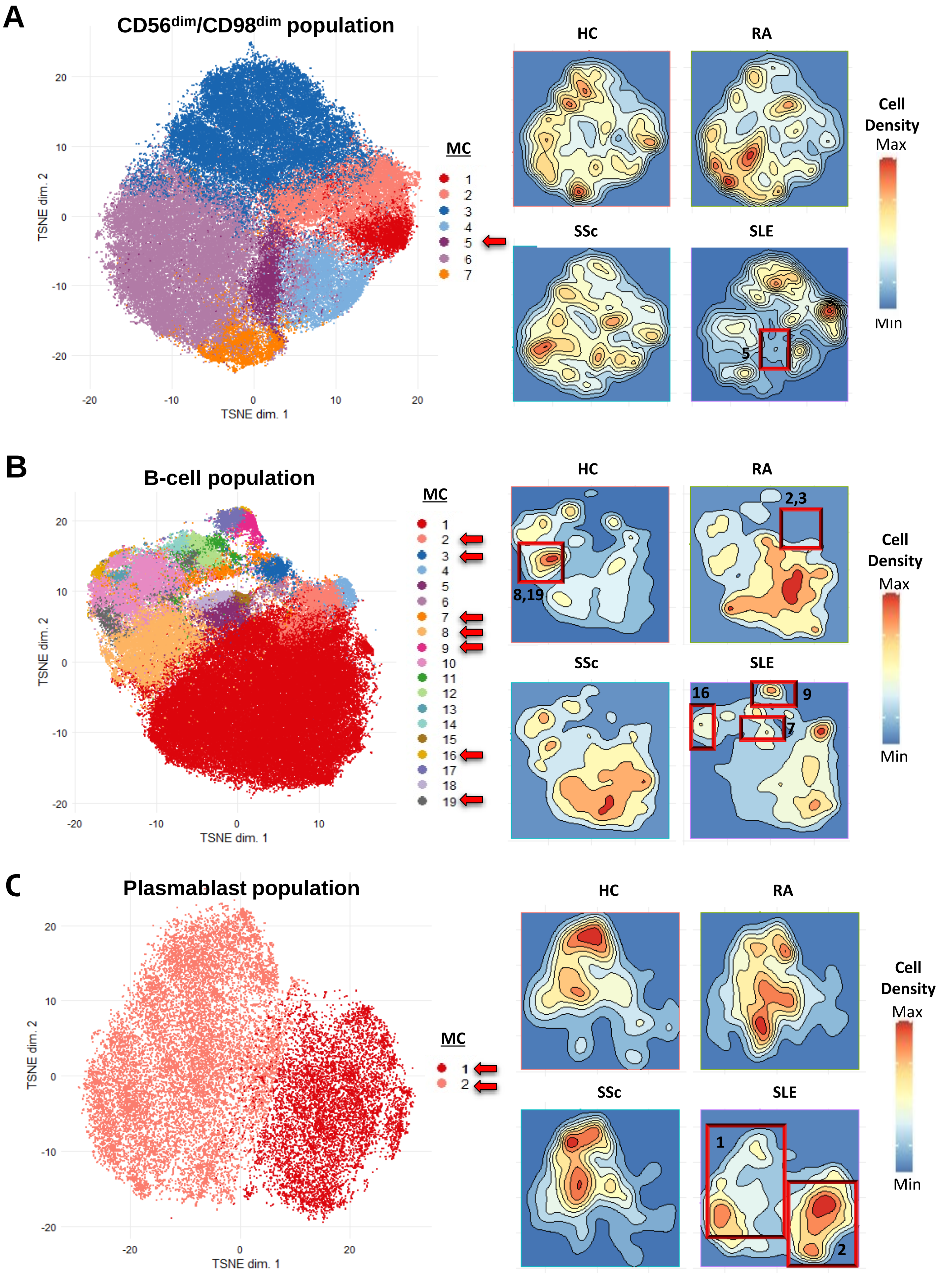

Supplementary Figure 15.

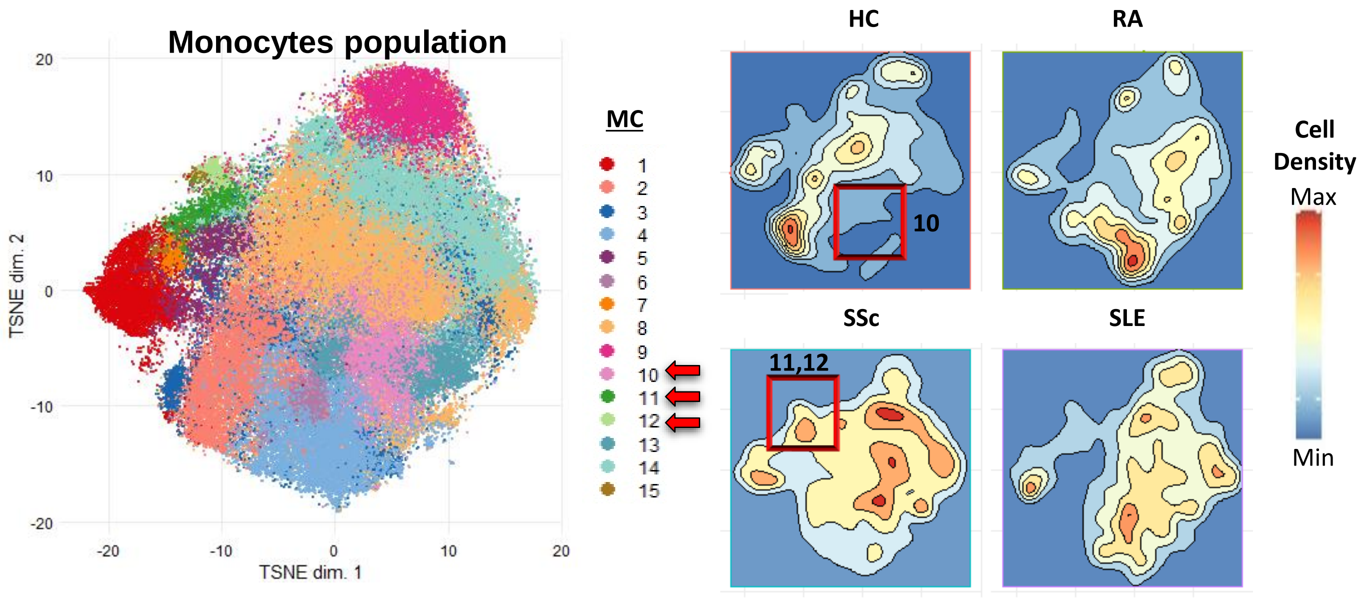

Supplementary Figure 16.
