# Supplementary material for: Comparative single-cell multiplex immunophenotyping of therapy-naive patients with rheumatoid arthritis, systemic sclerosis, and systemic lupus erythematosus shed light on disease-specific composition of the peripheral immune system
Source: Front Immunol. 2024 Apr 25;15:1376933. doi: 10.3389/fimmu.2024.1376933 (PMC11079270; doi:10.3389/fimmu.2024.1376933)
Supplement: Supplementary file 2 [file Table_2.docx]

| Characteristics | SSc patients (n=13) |
| --- | --- |
| Age, years | 63 (29-75) |
| female/male | 11/2 |
| disease subtype (dcSSc/lcSSc) | 9/4 |
| ANA positivity | 13/13 |
| Anti-Scl70 positivity | 11 |
| Anti-centromer positivity | 2 |
| Skin involvement | 13 |
| Skin score | 12 (5-19) |
| Raynaud phenomenon | 12 |
| Digital ulceration | 2 |
| Arthritis | 4 |
| Intersticial lung involvement | 5 |
| Pulmonary hypertension | 2 |
| GI involvement | 8 |

| Characteristics | RA patients (n=13) |
| --- | --- |
| Age, years | 57 (29-73) |
| female/male | 10/3 |
| anti-MCV positivity | 13 |
| RF positivity | 13 |
| DAS-28 score | 4.9 (4.0-5.7) |

**Supplementary Table 1: Demographic and clinical data of RA patients**

Data are expressed as numbers or median (range). RA, rheumatoid arthritis; anti-MCV, anti-mutated citrullianted vimentin; RF, rheumatoid factor; DAS-28, disease activity score-28

**Supplementary Table 2: Demographic and clinical data of SSc patients**

Data are expressed as numbers or median (range). SSc, systemic sclerosis; dcSSc, diffuse cutaneous systemic sclerosis; lcSSc, limited cutan systemic sclerosis; ANA, antinuclear antibody; GI, gastrointestinal; Skin score, modified Rodnan skin thickness score;

| Characteristics | SLE patients (n=13) |
| --- | --- |
| Age, years | 50 (20-72) |
| female/male | 13 |
| SLEDAI-2K | 11 (8-24) |
| ANA positivity | 13 |
| Anti-dsDNA positivity | 13 |
| Low complement | 8 |
| LA positivity | 7 |
| Coombs positivity | 4 |
| Arthritis/arthralgia | 10 |
| photosensitivity | 6 |
| Butterfly rush | 2 |
| Other skin lesion | 4 |
| Oral ulceration | 3 |
| pericarditis | 3 |
| pleuritis | 3 |
| Lupus nephritis | 1 |
| Interstitial lung involvement | 1 |
| vasculitis | 2 |
| Haemolytic anemia | 1 |
| lymphopenia | 4 |
| thrombocytopenia | 3 |

**Supplementary Table 3: Demographic and clinical data of SLE patients**

# Data are expressed as numbers or median (range). SLE, systemic lupus erythematosus; SLEDAI-2K, Systemic Lupus Erythematosus Disease Activity Index 2000; ANA, antinuclear antibody; anti-dsDNA, antibody to double-stranded deoxyribonucleic acid, LA, lupus anticoagulant test
